# Novel complex fuzzy distance measures with hesitance values and their applications in complex decision-making problems

**DOI:** 10.1038/s41598-024-64112-6

**Published:** 2024-06-20

**Authors:** Madad Khan, Safi Ullah, Muhammad Zeeshan, Ramsha Shafqat, Imen Kebaili, Tola Bekene Bedada, Saima Anis

**Affiliations:** 1 Department of Mathematics, COMSATS University Islamabad, Abbottabad Campus, Abbottabad, Pakistan; 2 Department of Mathematics, COMSATS University Islamabad, Islamabad Campus, Islamabad, Pakistan; 3 Department of Mathematics, The University of Agriculture, Dera Ismail Khan, Pakistan; 4 Department of Mathematics and Statistics, The University of Lahore, Sargodha, 40100 Pakistan; 5 Department of Physics, Faculty of Science, King Khalid University, P.O. Box 9004 Abha, Saudi Arabia; 6 Department of Mathematics, Wachemo University, Hosaina, Ethiopia

**Keywords:** Complex fuzzy sets, Complex fuzzy operations, Complex fuzzy distance measures, Decision-making algorithm., Engineering, Mathematics and computing

## Abstract

A complex fuzzy distance measure (CFDMs) plays a significant role in applications involving complex or high-dimensional data where traditional distance measures may not adequately capture the nuances of the data relationships. The significance of CFDMs lies in their ability to handle uncertainty, imprecision, and complexity in various domains. Numerous researchers introduced different concepts of CFDMs, yet these CFDMs fails to convey any information regarding the hesitancy degree associated with an element. The main objective of this paper is to introduce some new distance measures based on complex fuzzy sets, called complex fuzzy hesitance distance measure and complex fuzzy Euclidean Hesitance distance measure, which is the generalization of complex fuzzy normalized Hamming distance measure and complex fuzzy Euclidean distance measure. Some new operations and primay results are discussed in the environment of proposed CFDMs and complex fuzzy operations. Moreover, we discussed the applications of the proposed CFDMs in addressing decision-making problems. We introduced a new decision-making algorithm that integrates CFDMs into decision-making processes, providing a robust methodology for handling real-world complexities. Further, the comparative study of the proposed CFDMs is discussed with some existing CFDMs.

## Introduction

Fuzzy set theory^1^ is a mathematical framework developed by Lofti Zadeh in 1965 to deal with uncertainties and vagueness in data. Unlike traditional set theory, which only deals with binary membership (an element either belongs or does not belong to a set), the fuzzy set theory allows for degrees of membership, where an element can belong to a set to a certain degree or not at all. Fuzzy sets have many applications, particularly in areas where traditional set theory may not be applicable due to uncertainty or imprecision, such as in artificial intelligence, control systems, decision-making, and pattern recognition. Fuzzy sets have also been used in fields like .nance, medicine, and environmental science. Adlassning discussed applications of fuzzy set theory in medical diagnosis problems^2^. Pedrycz introduced the fundamental concepts of fuzzy sets theory^3^. They solved different real-world problems based on these fundamental concepts. Kabir and Papadopoulos discussed applications of FSs to safety and reliability engineering^4^. McBratney and Odeh proposed a decision-making method for applications in soil sciences based on fuzzy logic and fuzzy measurements^5^.

Intuitionistic fuzzy set (IFS) is the generalization of a fuzzy set introduced by Atanassov in 1986^6^. The IFS model is more significant than the FS model due to its structure. Intuitionistic fuzzy sets have been used in various applications, including decision-making^7^, pattern recognition, image processing, and data mining. They are beneficial in situations where uncertainty and ambiguity are present and where a more nuanced approach to membership and non-membership is required. Xiao defined a novel DM under the environment of IFSs^8^. He developed an algorithm for patternclassification based on the proposed intuitionistic fuzzy distance measure (IFDM). Gohain et al.^9^ proposed new IFDM. They discussed their applications to pattern recognition, clustering, and decision-making problems. Alkan and Kahraman introduced a new intuitionistic fuzzy multi-distance measure based on Euclidean and cosine DM^10^. They established a decision-making method for applications of waste disposal location selection. Zeng et al.^11^ developed a new IFDM is based on membership degree, non-membership degree, and the difference between membership and non-membership degree. They utilized the proposed DM for applications in pattern recognition problems. Patel et al.^12^ gave the notions of a 3D IFDM and discussed its applications in pattern recognition and decision-making problems. The concepts of nonlinear strict intuitionistic fuzzy distance and intuitionistic fuzzy similarity measure were introduced by Wu et al. in^13^. They study their applications in pattern recognition and medical diagnosis.

Yager gave the notion of Pythagorean fuzzy sets in^14^. PFS is the generalization of fuzzy and intuitionistic fuzzy sets. PFS can also be used to model imprecise and uncertain information in various applications, such as decision-making, image processing, and pattern recognition^15^. Ejegwa et al.^16^ defined a new tri-parametric distance and weighted tri-parametric distance under the environment of PFSs. They discussed their applications in pattern classification and disease diagnostic analysis. Wu et al.^17^ discussed some Pythagorean fuzzy similarity measuring methods based on the Pythagorean fuzzy distance measures (PFDMs). They utilized them for medical diagnosis and pattern recognition problems. Mahanta and Panda presented a novel DM under the environment of PFSs in^18^. They established a decision-making method based on the PFDM. Ejegwa and Awolola introduced some new PFDMs by incorporating the conventional parameters that describe PFSs^19^. They utilized the proposed DM in pattern recognition problems. Li et al.^20^ developed new DMs and similarity measures under the environment of PFSs. They presented its application to the selection of advertising platforms. Dutta et al.^21^ gave the concept of nonlinear PFDMs. They discussed their applications in problems of pattern recognition, medical diagnosis, and COVID-19 medicine selection. Yin et al.^22^ defined the notion of a new DM in the environment of Pythagorean fuzzy sets. They utilized them in solving algorithms of pattern recognition, medical diagnosis, and multi-criteria decision-making (MCDM) problems.

Fuzzy sets, intuitionistic fuzzy sets, and Pythagorean fuzzy sets, these three types of sets all other a more flexible and nuanced representation of uncertainty and imprecision than classical set theory and have been applied in decision-making^23^, pattern recognition, and image processing. However, these three models cannot handle two-dimensional problems. To discuss two-dimensional phenomena, Ramot et al.^24^ gave the idea of complex fuzzy sets (CFSs). A CFS is the generalization of a fuzzy set whose membership function is complex-valued, that is, its range is the unit disk in a complex plane. The complex-valued function describes the degree of membership of a member in the CFS using both magnitude and phase information. CFSs have found applications in various fields, such as image processing, pattern recognition, control systems, and decision-making^25,26^. They provide a powerful tool for modeling and analyzing complex systems with uncertainty and imprecision. Liu et al.^27^ discussed a decision-making method based on CFDi-Me and complex fuzzy entropy measures. Hu et al.^28^ introduced some DMs in the environment of CFSs. They discussed their applications to continuity problems. Zhang et al.^29^ developed a new CFDM based on the maximum operator. They proposed an algorithm for signal processing in the CFDMs environment. Zeeshan et al.^30^ defined new Di-Mes in the environment of CFSs. They constructed an algorithm for the high resemblance of a signal based on CFDMs. A new algorithm for signal processing methods in the environment of CFSs was proposed by Ma et al. in^31^. Khan et al.^32^ discussed a new method for identifying reference signals out of large signals under the environment of CFSs. Zeeshan et al.^33^ established a new method for identifying a reference signal based on the distance function of complex fuzzy soft sets. A new self-learning complex neuro-fuzzy system based on CFSs was developed by Li in^34^. They utilized them in the adaptive image noise-canceling process. Dai et al. introduced some series of interval-valued CFDMs and discussed different properties of these DMs. Hu et al.^35^ defined new DMs based on the orthogonality property of CFSs. They utilized the proposed DM in applications of signal processing. Cosine similarity measures between CFSs were proposed by Guo et al. in^36^. They applied the proposed cosine similarity measures to the robustness of complex fuzzy connectives and inference. Song et al.^37^ defined some new DMs based on interval-valued CFSs. They developed a new decision-making method in the environment of interval-valued CFSs. Some novel complex fuzzy ordered weighted distance measures (CFOWDMs) are introduced by Dai in^38^. He presented an illustrative example regarding the selected problem based on CFOWDMs. Al-Qudah and Hassan gave the concept of complex multi-fuzzy sets and complex multi-fuzzy distance measures^39^. They introduced the basic operations on complex multi-fuzzy sets.

The main objective of this paper is to introduce some new DMs based on CFSs, called as CFHDM and CFEHDM which, are the generalization of CFNHDM and CFEDM. Some new operations and basic results are discussed in the environment of proposed CFDMs and complex fuzzy operations. Moreover, we discussed the applications of the proposed CFDMs in addressing decision-making problems. We introduced a new decision-making algorithm integrating CFDMs into decision-making processes, providing a robust methodology for handling real-world complexities. Further, the comparative study of the proposed CFDMs is discussed with some existing CFDMs.

### Research gap

Further research is needed to establish a comprehensive theoretical framework for complex fuzzy distance measures, including investigating mathematical properties, axiomatic foundations, and formal characterization. Empirical validation and dynamic decision-making frameworks are also essential. Addressing these gaps will advance theoretical foundations, computational methodologies, empirical validation, interpretability, integration with decision support systems, and domain-specific applications of these measures.

### Motivation

Complex fuzzy hesitance distance measures are essential in decision-making, especially when decision-makers face uncertainty and vagueness in their preferences. These measures allow decision-makers to express their preferences in a more nuanced and flexible manner, capturing the complexity of decision-making processes accurately. They are particularly useful in multi-criteria decision analysis (MCDA) problems, where decisions need to be made based on multiple, often conflicting criteria. They also help decision-makers represent and manage uncertainty effectively by incorporating fuzzy logic principles. Furthermore, they provide a range of possible evaluations for each alternative, allowing decision-makers to convey uncertainty more effectively, leading to more robust decision outcomes.

### Contributions

Complex fuzzy hesitance distance measures are crucial in decision-making problems. They quantify hesitancy, providing insights into uncertainty or indecision associated with alternatives. These measures can be integrated into decision-making frameworks like MCDA or DSS, enabling more informed and reliable decisions in complex environments. They offer flexibility in representing preferences, accommodating various degrees of hesitancy and ambiguity, and can handle incomplete or imprecise information, enhancing decision models’ robustness.

## Some basic concepts

In this section, we will revise the basic notions of FS theory used through out the thesis.

### Definition 1

^1^ Let ${\mathcal {L}}$ be a nonempty set. A FS ${\mathbb {R}}$ is defined as

1 $$\begin{aligned} {\mathbb {R}} =\left\{ \left\langle r,\Im _{ {\mathbb {R}} }(r):r\in {\mathcal {L}} \right\rangle \right\} , \end{aligned}$$

where $\Im _{ {\mathbb {R}} }(r): {\mathcal {L}} \longrightarrow \left[ 0,1\right]$ denotes the truth grade of ${\mathbb {R}}$ for any element $r\in {\mathcal {L}}.$

### Definition 2

^6^ Let ${\mathcal {L}}$ be a nonempty set. An IFS ${\mathbb {R}}$ is defined as

2 $$\begin{aligned} {\mathbb {R}} =\left\{ \left( r,\left\langle \varepsilon _{ {\mathbb {R}} }(r),\beta _{ {\mathbb {R}} }(r)\right\rangle \right) :r\in {\mathcal {L}} \right\} , \end{aligned}$$

where $\varepsilon _{ {\mathbb {R}} }(r): {\mathcal {L}} \longrightarrow \left[ 0,1\right]$ and $\beta _{ {\mathbb {R}} }(r): {\mathcal {L}} \rightarrow [0,1]$ denote the truth grade and false grade, respectively, of ${\mathbb {R}}$ for any element $r\in {\mathcal {L}} ,$ and satisfied the condition

$$\begin{aligned} 0\le \left( \varepsilon _{ {\mathbb {R}} }(r)\right) +\left( \beta _{ {\mathbb {R}} }\left( r\right) \right) \le 1. \end{aligned}$$

### Definition 3

^24^ A CFS ${\mathbb {R}}$ is characterized by a grade value $\Im _{ {\mathbb {R}} }(x)=\ell _{ {\mathbb {R}} }(r)e^{iw_{ {\mathbb {R}} }(x)}$ on a universe of discourse ${\mathcal {L}} .$ In a CFS ${\mathbb {R}}$, the GV $\Im _{ {\mathbb {R}} }(r)$ of an element $r\in {\mathcal {L}} ,$ is determined by a complex-valued function rather than a simple grade value. In the grade value of CFS ${\mathbb {R}},$ the term $\ell _{ {\mathbb {R}} }(r)$ is said to be an amplitude term and $w_{ {\mathbb {R}} }(r)$ is called a phase term.

Note that Both these functions are real-valued and $\ell _{ {\mathbb {R}} }(r)\in [0,1].$

The Hesitance degree of CFS is denoted by $\hslash _{ {\mathbb {R}} }(x)$ and is given by $\hslash _{ {\mathbb {R}} }\left( x\right) =|\left( 1-\ell _{ {\mathbb {R}} }(x)\right) -\frac{1}{2\pi }\left( 2\pi -w_{ {\mathbb {R}} }(x)\right) |.$

Mathematically, it can be written as:

3 $$\begin{aligned} {\mathbb {R}}&=\{(r;\Im _{ {\mathbb {R}} }(r)):r\in {\mathcal {L}} \},\\&=\ell _{ {\mathbb {R}} }(r)e^{iw_{ {\mathbb {R}} }(r)}:r\in {\mathcal {L}}. \end{aligned}$$

### Definition 4

^29^ Let ${\mathbb {R}} _{n},$ $n=1,2,3,\ldots ,N$ be *N* CFS defined on ${\mathcal {L}}$ and $\Im _{ {\mathbb {R}} _{n}}(r)=\ell _{ {\mathbb {R}} _{n}}(r)e^{iw_{ {\mathbb {R}} _{n}}(r)}$ their grade values. The complex fuzzy (CF) Cartesian product of ${\mathbb {R}} _{n}$ represented by $\underset{i=1}{\overset{n}{\times }} {\mathbb {R}} _{i}= {\mathbb {R}} _{1}\times {\mathbb {R}} _{2}\times {\mathbb {R}} _{3}\times \cdots \times {\mathbb {R}} _{n},$ is defined by a function

4 $$\begin{aligned} \Im _{\underset{i=1}{\overset{n}{\times }} {\mathbb {R}} _{i}}\text { }(r)&=\text { }\ell _{\underset{i=1}{\overset{n}{\times }} {\mathbb {R}} _{i}}\text { }(r)e^{iw_{\underset{i=1}{\overset{n}{\times }} {\mathbb {R}} _{i}}(r)},\\&=\min (\ell _{ {\mathbb {R}} _{1}}(r_{1}),\ell _{ {\mathbb {R}} _{2}}(r_{2}),\ldots ,\ell _{ {\mathbb {R}} _{n}}(r_{n})) e^{i\min (w_{\text { } {\mathbb {R}} _{1}}(r_{1}),w_{\text { } {\mathbb {R}} _{2}}(r_{2}),\ldots ,w_{\text { } {\mathbb {R}} _{n}}(r_{n}))}. \end{aligned}$$

### Definition 5

^24^ Let ${\mathbb {R}} _{1}$ and ${\mathbb {R}} _{2}$ be two CFSs on ${\mathcal {L}} ,$ and $\Im _{ {\mathbb {R}} _{1}}(r)=\ell _{ {\mathbb {R}} _{1}}(r)e^{iw_{ {\mathbb {R}} _{1}}(r)}$ and $\Im _{ {\mathbb {R}} _{2}}(r)=\ell _{ {\mathbb {R}} _{2}}(r)e^{iw_{ {\mathbb {R}} _{2}}(r)}$ their grade values, respectively. The CF intersection of ${\mathbb {R}} _{1}$ and ${\mathbb {R}} _{2}$ is denoted by ${\mathbb {R}} _{1}\Cap {\mathbb {R}} _{2}$, and is defined by a function

5 $$\begin{aligned} {\mathbb {R}} _{1}\Cap {\mathbb {R}} _{2}&=\ell _{ {\mathbb {R}} _{1}}(r)e^{iw_{ {\mathbb {R}} _{1}}(r)}\text { }\Cap \ell _{ {\mathbb {R}} _{2}}(r)e^{iw_{ {\mathbb {R}} _{2}}(r)},\\&=\min \left[ \ell _{ {\mathbb {R}} _{1}}(r),\ell _{ {\mathbb {R}} _{2}}(r)\right] e^{i\min \left[ w_{ {\mathbb {R}} _{1}}(r),w_{ {\mathbb {R}} _{2}}(r)\right] }. \end{aligned}$$

### Definition 6

^24^ Let ${\mathbb {R}} _{1}$ and ${\mathbb {R}} _{2}$ be two CFSs on ${\mathcal {L}} ,$ and $\Im _{ {\mathbb {R}} _{1}}(r)=\ell _{ {\mathbb {R}} _{1}}(r)e^{iw_{ {\mathbb {R}} _{1}}(r)}$ and $\Im _{ {\mathbb {R}} _{2}}(r)=\ell _{ {\mathbb {R}} _{2}}(r)e^{iw_{ {\mathbb {R}} _{2}}(r)}$ their grade values, respectively. The CF union of ${\mathbb {R}} _{1}$ and ${\mathbb {R}} _{2}$ is denoted by ${\mathbb {R}} _{1}\Cup {\mathbb {R}} _{2}$, and is defined by a function

6 $$\begin{aligned} {\mathbb {R}} _{1}\Cup {\mathbb {R}} _{2}&=\ell _{ {\mathbb {R}} _{1}}(r)e^{iw_{ {\mathbb {R}} _{1}}(r)}\Cup \ell _{ {\mathbb {R}} _{2}}(r)e^{iw_{ {\mathbb {R}} _{2}}(r)},\\&=\max \left[ \ell _{ {\mathbb {R}} _{1}}(r),\ell _{ {\mathbb {R}} _{2}}(r)\right] e^{i\max \left[ w_{ {\mathbb {R}} _{1}}(r),w_{ {\mathbb {R}} _{2}}(r)\right] }. \end{aligned}$$

### Definition 7

^24^ Let ${\mathbb {R}}$ be a CFS on ${\mathcal {L}},$ and $\Im _{ {\mathbb {R}} }(r)=\ell _{ {\mathbb {R}} }(r)e^{iw_{ {\mathbb {R}} }(r)}$ its grade value. The CF complement of a CFS ${\mathbb {R}}$ is expressed by ${\mathbb {R}} ^{c}$, and is given by

7 $$\begin{aligned} {\mathbb {R}} ^{c}&=\ell _{ {\mathbb {R}} ^{c}}(r)e^{iw_{ {\mathbb {R}} ^{c}}(r)}\\&=\left[ 1-\ell _{ {\mathbb {R}} }(r)\right] e^{iw_{ {\mathbb {R}} }(r)}. \end{aligned}$$

### Theorem 1

For any three CFSs ${\mathbb {R}} _{1},$ ${\mathbb {R}} _{2}$ and ${\mathbb {R}} _{3}$ on a universe of discourse ${\mathcal {L}} ,$ the standard intersection, union, and complement satisfy the following:

(i).: $$ {\mathbb {R}} _{1}\Cap {\mathbb {R}} _{2}= {\mathbb {R}} _{2}\Cap {\mathbb {R}} _{1},$$
(ii).: $$ {\mathbb {R}} _{1}\Cup {\mathbb {R}} _{2}= {\mathbb {R}} _{2}\Cup {\mathbb {R}} _{1},$$
(iii).: $$ {\mathbb {R}} _{1}\Cap ( {\mathbb {R}} _{2}\Cap {\mathbb {R}} _{3})=( {\mathbb {R}} _{1}\Cap {\mathbb {R}} _{2})\Cap {\mathbb {R}} _{3},$$
(iv).: $$ {\mathbb {R}} _{1}\Cup ( {\mathbb {R}} _{2}\Cup {\mathbb {R}} _{3})=( {\mathbb {R}} _{1}\Cup {\mathbb {R}} _{2})\Cup {\mathbb {R}} _{3},$$
(v).: $$ {\mathbb {R}} _{1}\Cap ( {\mathbb {R}} _{2}\Cup {\mathbb {R}} _{3})=( {\mathbb {R}} _{1}\Cap {\mathbb {R}} _{2})\Cup ( {\mathbb {R}} _{1}\Cap {\mathbb {R}} _{3}),$$
(vi).: $$ {\mathbb {R}} _{1}\Cup ( {\mathbb {R}} _{2}\Cap {\mathbb {R}} _{3})=( {\mathbb {R}} _{1}\Cup {\mathbb {R}} _{2})\Cap ( {\mathbb {R}} _{1}\Cup {\mathbb {R}} _{3}),$$
(vii).: $$( {\mathbb {R}} _{1}\Cap {\mathbb {R}} _{2})^{c}= {\mathbb {R}} _{2}^{c}\Cup {\mathbb {R}} _{1}^{c},$$
(viii).: $$( {\mathbb {R}} _{1}\Cup {\mathbb {R}} _{2})^{c}= {\mathbb {R}}_{2}^{c}\Cap {\mathbb {R}} _{1}^{c}.$$

### Proof

(i). Let ${\mathbb {R}} _{1}$ and ${\mathbb {R}} _{2}$ be two CFSs and $\Im _{ {\mathbb {R}} _{1}}(r)=\ell _{ {\mathbb {R}} _{1}}(r)e^{iw_{ {\mathbb {R}} _{1}}(r)},$ $\Im _{ {\mathbb {R}} _{2}}(r)=$ $\ell _{ {\mathbb {R}} _{2}}(r)$ $e^{iw_{ {\mathbb {R}} _{2}}(r)}$ represent their membership functions, respectively. Then

$$\begin{aligned} {\mathbb {R}} _{1}\Cap {\mathbb {R}} _{2}&=\ell _{ {\mathbb {R}} _{1}}(r)e^{iw_{ {\mathbb {R}} _{1}}(r)}\Cap \ell _{ {\mathbb {R}} _{2}}(r)e^{iw_{ {\mathbb {R}} _{2}}(r)}\\&=\min \left[ \ell _{ {\mathbb {R}} _{1}}(r),\ell _{ {\mathbb {R}} _{2}}(r)\right] \cdot e^{i\min \left[ w_{ {\mathbb {R}} _{1}}(r),w_{ {\mathbb {R}} _{2}}(r)\right] },\\&=\min \left[ \ell _{ {\mathbb {R}} _{2}}(r),\ell _{ {\mathbb {R}} _{1}}(r)\right] \cdot e^{i\min \left[ w_{ {\mathbb {R}} _{2}}(r),w_{ {\mathbb {R}} _{1}}(r)\right] },\\&=\ell _{ {\mathbb {R}} _{2}}(r)e^{iw_{ {\mathbb {R}} _{2}}(r)}\Cap \ell _{ {\mathbb {R}} _{1}}(r)e^{iw_{ {\mathbb {R}} _{1}}(r)},\\&= {\mathbb {R}} _{2}\Cap {\mathbb {R}} _{1}. \end{aligned}$$

(ii). It is easy to prove.

(iii). Let ${\mathbb {R}} _{1},$ ${\mathbb {R}} _{3},$ and ${\mathbb {R}} _{3}$ be three CFSs and $\Im _{ {\mathbb {R}} _{1}}(r)=\ell _{ {\mathbb {R}} _{1}}(r)e^{iw_{ {\mathbb {R}} _{1}}(r)},$ $\Im _{ {\mathbb {R}} _{2}}(r)=$ $\ell _{ {\mathbb {R}} _{2}}(r)$ $e^{iw_{ {\mathbb {R}} _{2}}(r)}$ and $\Im _{ {\mathbb {R}} _{3}}(r)=$ $\ell _{ {\mathbb {R}} _{3}}(r)$ $e^{iw_{ {\mathbb {R}} _{3}}(r)}$ represent their membership functions, respectively. Then

$$\begin{aligned} {\mathbb {R}} _{1}\Cap ( {\mathbb {R}} _{2}\Cap {\mathbb {R}} _{3})&=\ell _{ {\mathbb {R}} _{1}}(r)e^{iw_{ {\mathbb {R}} _{1}}(r)}\Cap \ell _{ {\mathbb {R}} _{2}\Cap {\mathbb {R}} _{3}}(r)e^{iw_{ {\mathbb {R}} _{2}\Cup {\mathbb {R}} _{3}}(r)}\\&=\min \left[ \ell _{ {\mathbb {R}} _{1}}(r),\min \left( \ell _{ {\mathbb {R}} _{2}}(r),\ell _{ {\mathbb {R}} _{3}}(r)\right) \right]&e^{i\min \left[ w_{ {\mathbb {R}} _{1}}(r),\min \left( w_{ {\mathbb {R}} _{2}}(r),w_{ {\mathbb {R}} _{3}}(r)\right) \right] },\\&=\min \left[ \ell _{ {\mathbb {R}} _{1}}(r),\ell _{ {\mathbb {R}} _{2}}(r),\ell _{ {\mathbb {R}} _{3}}(r)\right]&e^{i\min \left[ w_{ {\mathbb {R}} _{1}}(r),w_{ {\mathbb {R}} _{2}}(r),w_{ {\mathbb {R}} _{3}}(r)\right] },\\&=\min \left[ \min (\ell _{ {\mathbb {R}} _{1}}(r),\ell _{ {\mathbb {R}} _{2}}(r)),\ell _{ {\mathbb {R}} _{3}}(r)\right] \\&=e^{i\min \left[ \min (w_{ {\mathbb {R}} _{1}}(r),w_{ {\mathbb {R}} _{2}}(r)),w_{ {\mathbb {R}} _{3}}(r)\right] },\\&=\ell _{ {\mathbb {R}} _{1}\Cap {\mathbb {R}} _{2}}(r)e^{iw_{ {\mathbb {R}} _{1}\Cap {\mathbb {R}} _{2}}(r)}\Cap \ell _{ {\mathbb {R}} _{3}}(r)e^{iw_{ {\mathbb {R}} _{3}}(r)},\\&=( {\mathbb {R}} _{1}\Cap {\mathbb {R}} _{2})\Cap {\mathbb {R}} _{3}. \end{aligned}$$

(iv). It is easy to prove.

(v). Let ${\mathbb {R}} _{1},$ ${\mathbb {R}} _{3},$ and ${\mathbb {R}} _{3}$ be three CFSs and $\Im _{ {\mathbb {R}} _{1}}(r)=\ell _{ {\mathbb {R}} _{1}}(r)e^{iw_{ {\mathbb {R}} _{1}}(r)},$ $\Im _{ {\mathbb {R}} _{2}}(r)=$ $\ell _{ {\mathbb {R}} _{2}}(r)$ $e^{iw_{ {\mathbb {R}} _{2}}(r)}$ and $\Im _{ {\mathbb {R}} _{3}}(r)=$ $\ell _{ {\mathbb {R}} _{3}}(r)$ $e^{iw_{ {\mathbb {R}} _{3}}(r)}$ represent their membership functions, respectively. Then,

7 $$\begin{aligned} {\mathbb {R}} _{1}\Cap ( {\mathbb {R}} _{2}\Cup {\mathbb {R}} _{3})&=\ell _{ {\mathbb {R}} _{1}}(r)e^{iw_{ {\mathbb {R}} _{1}}(r)}\Cap \ell _{ {\mathbb {R}} _{2}\Cup {\mathbb {R}} _{3}}(r)e^{iw_{ {\mathbb {R}} _{2}\Cup {\mathbb {R}} _{3}}(r)},\\&=\min \left[ \ell _{ {\mathbb {R}} _{1}}(r),\max \left( \ell _{ {\mathbb {R}} _{2}}(r),\ell _{ {\mathbb {R}} _{3}}(r)\right) \right]&e^{i\min \left[ w_{ {\mathbb {R}} _{1}}(r),\max \left( w_{ {\mathbb {R}} _{2}}(r),w_{ {\mathbb {R}} _{3}}(r)\right) \right] }. \end{aligned}$$

8 $$\begin{aligned} ( {\mathbb {R}} _{1}\Cap {\mathbb {R}} _{2})\Cup ( {\mathbb {R}} _{1}\Cap {\mathbb {R}} _{3})&=\ell _{ {\mathbb {R}} _{1}\Cap {\mathbb {R}} _{2}}(r)e^{iw_{ {\mathbb {R}} _{1}\Cap {\mathbb {R}} _{2}}(r)}\Cup \ell _{ {\mathbb {R}} _{1}\Cap {\mathbb {R}} _{3}}(r)e^{iw_{ {\mathbb {R}} _{1}\Cap {\mathbb {R}} _{3}}(r)},\\&=\max \left[ \min \left( \ell _{ {\mathbb {R}} _{1}}(r),\ell _{ {\mathbb {R}} _{2}}(r)\right) ,\min \left( \ell _{ {\mathbb {R}} _{1}}(r),\ell _{ {\mathbb {R}} _{3}}(r)\right) \right] \\&\quad e^{i\max \left[ \min \left( w_{ {\mathbb {R}} _{1}}(r),w_{ {\mathbb {R}} _{2}}(r)\right) ,\min \left( w_{ {\mathbb {R}} _{1}}(r),w_{ {\mathbb {R}} _{3}}(r)\right) \right] }. \end{aligned}$$

To prove the distributive law of intersection over union, we take some cases.

Case 1. If $\ell _{ {\mathbb {R}} _{1}}(r)\le \ell _{ {\mathbb {R}} _{2}}(r)\le \ell _{ {\mathbb {R}} _{3}}(r)$ and $w_{ {\mathbb {R}} _{1}}(r)\le w_{ {\mathbb {R}} _{2}}(r)\le w_{ {\mathbb {R}} _{3}}(r)$ then (7) and (8) implies that

9 $$\begin{aligned} {\mathbb {R}} _{1}\Cap ( {\mathbb {R}} _{2}\Cup {\mathbb {R}} _{3})&=\min \left[ \ell _{ {\mathbb {R}} _{1}}(r),\max \left( \ell _{ {\mathbb {R}} _{2}}(r),\ell _{ {\mathbb {R}} _{3}}(r)\right) \right] e^{i\min \left[ w_{ {\mathbb {R}} _{1}}(r),\max \left( w_{ {\mathbb {R}} _{2}}(r),w_{ {\mathbb {R}} _{3}}(r)\right) \right] }\\&=\min \left[ \ell _{ {\mathbb {R}} _{1}}(r),\ell _{ {\mathbb {R}} _{3}}(r)\right] e^{i\min \left[ w_{ {\mathbb {R}} _{1}}(r),w_{ {\mathbb {R}} _{3}}(r)\right] },\\&=\ell _{ {\mathbb {R}} _{1}}(r) \cdot e^{iw_{ {\mathbb {R}} _{1}}(r)}. \end{aligned}$$

10 $$\begin{aligned} ( {\mathbb {R}} _{1}\Cap {\mathbb {R}} _{2})\Cup ( {\mathbb {R}} _{1}\Cap {\mathbb {R}} _{3})&=\max \left[ \min \left( \ell _{ {\mathbb {R}} _{1}}(r),\ell _{ {\mathbb {R}} _{2}}(r)\right) ,\min \left( \ell _{ {\mathbb {R}} _{1}}(r),\ell _{ {\mathbb {R}} _{3}}(r)\right) \right] \\&\quad e^{i\max \left[ \min \left( w_{ {\mathbb {R}} _{1}}(r),w_{ {\mathbb {R}} _{2}}(r)\right) ,\min \left( w_{ {\mathbb {R}} _{1}}(r),w_{ {\mathbb {R}} _{3}}(r)\right) \right] },\\&=\max \left[ \ell _{ {\mathbb {R}} _{1}}(r),\ell _{ {\mathbb {R}} _{1}}(r)\right] e^{i\max \left[ w_{ {\mathbb {R}} _{1}}(r),w_{ {\mathbb {R}} _{1}}(r)\right] },\\&=\ell _{ {\mathbb {R}} _{1}}(r) \cdot e^{iw_{ {\mathbb {R}} _{1}}(r)}. \end{aligned}$$

From (9) and (10) we have

$$\begin{aligned} {\mathbb {R}} _{1}\Cap ( {\mathbb {R}} _{2}\Cup {\mathbb {R}} _{3})=( {\mathbb {R}} _{1}\Cap {\mathbb {R}} _{2})\Cup ( {\mathbb {R}} _{1}\Cap {\mathbb {R}} _{3}) \end{aligned}$$

Case 1. If $\ell _{ {\mathbb {R}} _{3}}(r)\le \ell _{ {\mathbb {R}} _{2}}(r)\le \ell _{ {\mathbb {R}} _{1}}(r)$ and $w_{ {\mathbb {R}} _{3}}(r)\le w_{ {\mathbb {R}} _{2}}(r)\le w_{ {\mathbb {R}} _{1}}(r)$ then (7) and (8) implies that

11 $$\begin{aligned} {\mathbb {R}} _{1}\Cap ( {\mathbb {R}} _{2}\Cup {\mathbb {R}} _{3})&=\min \left[ \ell _{ {\mathbb {R}} _{1}}(r),\max \left( \ell _{ {\mathbb {R}} _{2}}(r),\ell _{ {\mathbb {R}} _{3}}(r)\right) \right] e^{i\min \left[ w_{ {\mathbb {R}} _{1}}(r),\max \left( w_{ {\mathbb {R}} _{2}}(r),w_{ {\mathbb {R}} _{3}}(r)\right) \right] }\\&=\min \left[ \ell _{ {\mathbb {R}} _{1}}(r),\ell _{ {\mathbb {R}} _{2}}(r)\right] e^{i\min \left[ w_{ {\mathbb {R}} _{1}}(r),w_{ {\mathbb {R}} _{2}}(r)\right] },\\&=\ell _{ {\mathbb {R}} _{2}}(r) \cdot e^{iw_{ {\mathbb {R}} _{2}}(r)}. \end{aligned}$$

12 $$\begin{aligned} ( {\mathbb {R}} _{1}\Cap {\mathbb {R}} _{2})\Cup ( {\mathbb {R}} _{1}\Cap {\mathbb {R}} _{3})&=\max \left[ \min \left( \ell _{ {\mathbb {R}} _{1}}(r),\ell _{ {\mathbb {R}} _{2}}(r)\right) ,\min \left( \ell _{ {\mathbb {R}} _{1}}(r),\ell _{ {\mathbb {R}} _{3}}(r)\right) \right] \nonumber \\&\quad e^{i\max \left[ \min \left( w_{ {\mathbb {R}} _{1}}(r),w_{ {\mathbb {R}} _{2}}(r)\right) ,\min \left( w_{ {\mathbb {R}} _{1}}(r),w_{ {\mathbb {R}} _{3}}(r)\right) \right] },\nonumber \\&=\max \left[ \ell _{ {\mathbb {R}} _{2}}(r),\ell _{ {\mathbb {R}} _{3}}(r)\right] e^{i\max \left[ w_{ {\mathbb {R}} _{2}}(r),w_{ {\mathbb {R}} _{3}}(r)\right] },\nonumber \\&=\ell _{ {\mathbb {R}} _{2}}(r) \cdot e^{iw_{ {\mathbb {R}} _{2}}(r)}. \end{aligned}$$

From (11) and (12), we have

$$\begin{aligned} {\mathbb {R}} _{1}\Cap ( {\mathbb {R}} _{2}\Cup {\mathbb {R}} _{3})=( {\mathbb {R}} _{1}\Cap {\mathbb {R}} _{2})\Cup ( {\mathbb {R}} _{1}\Cap {\mathbb {R}} _{3}). \end{aligned}$$

The proof is similar for other cases.

(vii). Let ${\mathbb {R}} _{1}$ and ${\mathbb {R}} _{2}$ be two CFSs and $\Im _{ {\mathbb {R}} _{1}}(r)=\ell _{ {\mathbb {R}} _{1}}(r)e^{iw_{ {\mathbb {R}} _{1}}(r)},$ $\Im _{ {\mathbb {R}} _{2}}(r)=$ $\ell _{ {\mathbb {R}} _{2}}(r)$ $e^{iw_{ {\mathbb {R}} _{2}}(r)}$ represent their membership functions, respectively. Then

$$\begin{aligned} ( {\mathbb {R}} _{1}\Cap {\mathbb {R}} _{2})^{c}&=\ell _{( {\mathbb {R}} _{1}\Cap {\mathbb {R}} _{2})^{c}}(r)e^{iw_{( {\mathbb {R}} _{1}\Cap {\mathbb {R}} _{2})^{c}}(r)},\\&=(1-\min (\ell _{ {\mathbb {R}} _{1}}(r),\ell _{ {\mathbb {R}} _{2}}(r)) \cdot e^{i(2\pi -\min (w_{ {\mathbb {R}} _{1}}(r),w_{ {\mathbb {R}} _{2}}(r)))},\\&=\max (1-\ell _{ {\mathbb {R}} _{1}}(r),1-\ell _{ {\mathbb {R}} _{2}}(r))e^{i\max (2\pi -w_{ {\mathbb {R}} _{1}}(r),2\pi -w_{ {\mathbb {R}} _{2}}(r)))},\\&=\max (\ell _{ {\mathbb {R}} _{1}^{c}}(r),\ell _{ {\mathbb {R}} _{2}^{c}}(r))e^{i\max (w_{ {\mathbb {R}} _{1}^{c}}(r),w_{ {\mathbb {R}} _{2}^{c}}(r)))},\\&= {\mathbb {R}} _{1}^{c}\Cup {\mathbb {R}} _{2}^{c}. \end{aligned}$$

$$\square $$

### Definition 8

^29^ Let ${\mathbb {R}} _{1}$ and ${\mathbb {R}} _{2}$ be two CFSs on ${\mathcal {L}} ,$ and $\Im _{ {\mathbb {R}} _{1}}(r)=\ell _{ {\mathbb {R}} _{1}}(r)e^{iw_{ {\mathbb {R}} _{1}}(r)}$ and $\Im _{ {\mathbb {R}} _{2}}(r)=\ell _{ {\mathbb {R}} _{2}}(r)e^{iw_{ {\mathbb {R}} _{2}}(r)}$ their grade values, respectively. The CF product of ${\mathbb {R}} _{1}$ and ${\mathbb {R}} _{2}$ is denoted by ${\mathbb {R}} _{1}\circ {\mathbb {R}} _{2}$, and is defined by a function

$$\begin{aligned} {\mathbb {R}} _{1}\circ {\mathbb {R}} _{2}&=\ell _{ {\mathbb {R}} _{1}}(r)e^{iw_{ {\mathbb {R}} _{1}}(r)}\circ \ell _{ {\mathbb {R}} _{2}}(r)e^{iw_{ {\mathbb {R}} _{2}}(r)},\\&=\left[ \ell _{ {\mathbb {R}} _{1}}(r).\ell _{ {\mathbb {R}} _{2}}(r)\right] e^{i2\pi \left[ \frac{w_{ {\mathbb {R}} _{1}}(r)}{2\pi }.\frac{w_{ {\mathbb {R}} _{2}}(r)}{2\pi }\right] }. \end{aligned}$$

### Proposition 1

^29^ The CF product on ${\mathbb {R}} ( {\mathcal {L}} )$ is a t-norm.

### Definition 9

^29^ Let ${\mathbb {R}} _{1}$ and ${\mathbb {R}} _{2}$ be two CFSs on ${\mathcal {L}} ,$ and $\Im _{ {\mathbb {R}} _{1}}(r)=\ell _{ {\mathbb {R}} _{1}}(r)e^{iw_{ {\mathbb {R}} _{1}}(r)}$ and $\Im _{ {\mathbb {R}} _{2}}(r)=\ell _{ {\mathbb {R}} _{2}}(r)e^{iw_{ {\mathbb {R}} _{2}}(r)}$ their grade values, respectively. The CF probabilistic sum of ${\mathbb {R}} _{1}$ and ${\mathbb {R}} _{2}$ is denoted by ${\mathbb {R}} _{1}\mp {\mathbb {R}} _{2}$, and is defined by a function

$$\begin{aligned} {\mathbb {R}} _{1}\mp {\mathbb {R}} _{2}&=\ell _{ {\mathbb {R}} _{1}}(r)e^{iw_{ {\mathbb {R}} _{1}}(r)}\mp \ell _{ {\mathbb {R}} _{2}}(r)e^{iw_{ {\mathbb {R}} _{2}}(r)},\\&=\left[ \ell _{ {\mathbb {R}} _{1}}(r)\text { }+\text { }\ell _{ {\mathbb {R}} _{2}}(r)\text { }-\text { }\ell _{ {\mathbb {R}} _{1}}\text { }(r).\text { }\ell _{ {\mathbb {R}} _{2}}(r)\right] e^{i2\pi \left[ \frac{w_{ {\mathbb {R}} _{1}}\text { }(r)}{2\pi }+\frac{w_{ {\mathbb {R}} _{2}}\text { }(r)}{2\pi }-\frac{w_{ {\mathbb {R}} _{1}}\text { }(r)}{2\pi }.\frac{w_{ {\mathbb {R}} _{2}}\text { }(r)}{2\pi }\right] }. \end{aligned}$$

### Proposition 2

^29^ The CF probabilistic sum on ${\mathbb {R}} ( {\mathcal {L}} )$ is an s-norm.

### Definition 10

^29^ Let ${\mathbb {R}} _{1}$ and ${\mathbb {R}} _{2}$ be two CFSs on ${\mathcal {L}} ,$ and $\Im _{ {\mathbb {R}} _{1}}(r)=\ell _{ {\mathbb {R}} _{1}}(r)e^{iw_{ {\mathbb {R}} _{1}}(r)}$ and $\Im _{ {\mathbb {R}} _{2}}(r)=\ell _{ {\mathbb {R}} _{2}}(r)e^{iw_{ {\mathbb {R}} _{2}}(r)}$ their grade values, respectively. The CF bold sum of ${\mathbb {R}} _{1}$ and ${\mathbb {R}} _{2}$ is denoted by ${\mathbb {R}} _{1}\oplus {\mathbb {R}} _{2}$, and is defined by a function

$$\begin{aligned} {\mathbb {R}} _{1}\oplus {\mathbb {R}} _{2}&=\ell _{ {\mathbb {R}} _{1}}(r)e^{iw_{ {\mathbb {R}} _{1}}(r)}\oplus \ell _{ {\mathbb {R}} _{2}}(r)e^{iw_{ {\mathbb {R}} _{2}}(r)},\\&=\min [1,\ell _{ {\mathbb {R}} _{1}}(r)+\ell _{ {\mathbb {R}} _{2}}(r)]e^{i\min [2\pi ,w_{ {\mathbb {R}} _{1}}(r)+w_{ {\mathbb {R}} _{2}}(r)]}. \end{aligned}$$

### Proposition 3

^29^ The CF bold sum on ${\mathbb {R}} ( {\mathcal {L}} )$ is an s-norm.

### Definition 11

^29^ Let ${\mathbb {R}} _{1}$ and ${\mathbb {R}} _{2}$ be two CFSs on ${\mathcal {L}} ,$ and $\Im _{ {\mathbb {R}} _{1}}(r)=\ell _{ {\mathbb {R}} _{1}}(r)e^{iw_{ {\mathbb {R}} _{1}}(r)}$ and $\Im _{ {\mathbb {R}} _{2}}(r)=\ell _{ {\mathbb {R}} _{2}}(r)e^{iw_{ {\mathbb {R}} _{2}}(r)}$ their grade values, respectively. The CF bold intersection of ${\mathbb {R}} _{1}$ and ${\mathbb {R}} _{2}$ is denoted by ${\mathbb {R}} _{1}\ominus {\mathbb {R}} _{2}$, and is defined by a function

$$\begin{aligned} {\mathbb {R}} _{1}\ominus {\mathbb {R}} _{2}&=\ell _{ {\mathbb {R}} _{1}}(r)e^{iw_{ {\mathbb {R}} _{1}}(r)}\ominus \ell _{ {\mathbb {R}} _{2}}(r)e^{iw_{ {\mathbb {R}} _{2}}(r)},\\&=\max [0,\ell _{ {\mathbb {R}} _{1}}(r)+\ell _{ {\mathbb {R}} _{2}}(r)-1] e^{i\max [0,w_{ {\mathbb {R}} _{1}}(r)+w_{ {\mathbb {R}} _{2}}(r)-2\pi ]}. \end{aligned}$$

### Proposition 4

^29^ The CF bold intersection on ${\mathbb {R}} ( {\mathcal {L}} )$ is a t-norm.

### Distance measures (DMs)

Many researchers proposed various DMs and used them in different real-life problems. The DMs based on CFSs are useful mathematical technique in the algorithm of De-Ma and signals and systems. DMs of CFSs are used in different real-world problems. In the following some DMs like Zhang DM, Normalized Hamming DM, Hamming DM, Euclidean DM, and Normalized Euclidean DM are presented.

### Zhang DM

#### Definition 12

^29^ A Zhang DM of CFSs is a function $d_{Z}: {\mathbb {R}} ({\mathcal {L}} )\times {\mathbb {R}} ( {\mathcal {L}})\rightarrow [0,1]$ with the properties: for any ${\mathbb {R}}_{1}, {\mathbb {R}} _{2}, {\mathbb {R}} _{3}\in {\mathbb {R}} ( {\mathcal {L}} )($collection of CFSs)

(i).: $$0\le d_{Z}( {\mathbb {R}} _{1},{\mathbb {R}}_{2})\le 1,$$
(ii).: $$d_{Z}( {\mathbb {R}} _{1},{\mathbb {R}} _{2})=0 \Longleftrightarrow {\mathbb {R}} _{1}= {\mathbb {R}}_{2},$$
(iii).: $$d_{Z}( {\mathbb {R}} _{1},{\mathbb {R}} _{2})=d_{Z}({\mathbb {R}} _{2},{\mathbb {R}} _{1}),$$
(iv).: $$d_{Z}( {\mathbb {R}} _{1},{\mathbb {R}} _{3})\le d_{Z}({\mathbb {R}} _{1},{\mathbb {R}} _{2})+d_{Z}( {\mathbb {R}} _{2},{\mathbb {R}}_{3}).$$

The Zhang DM is defined as:

13 $$\begin{aligned} d_{Z}( {\mathbb {R}} _{1},{\mathbb {R}} _{2})=\max \left[ \underset{r\in {\mathcal {L}} }{\sup }|\ell _{ {\mathbb {R}} _{1}}(r)-\ell _{ {\mathbb {R}} _{2}}(r)|,\frac{1}{2\pi }\underset{r\in {\mathcal {L}} }{\sup }|w_{ {\mathbb {R}} _{1}}(r)-w_{ {\mathbb {R}} _{2}}(r)|\right] . \end{aligned}$$

#### Theorem 2

^29^ The function $d_{Z}$ defined by Eq. (13) is a distance function (D-function) of CFSs on${\mathcal {L}}.$

### Hamming DM

#### Definition 13

^40^ A Hamming DM of CFSs is a function $d_{H}: {\mathbb {R}} ( {\mathcal {L}} )\times {\mathbb {R}} ( {\mathcal {L}} )\rightarrow [0,1]$ with the properties: for any ${\mathbb {R}} _{1}, {\mathbb {R}} _{2}, {\mathbb {R}} _{3}\in {\mathbb {R}} ( {\mathcal {L}} )($collection of CFSs)

(i).: $$0\le d_{H}( {\mathbb {R}} _{1},{\mathbb {R}} _{2})\le 1,$$
(ii).: $$d_{H}( {\mathbb {R}} _{1},{\mathbb {R}} _{2})=0 \Longleftrightarrow {\mathbb {R}} _{1}= {\mathbb {R}} _{2},$$
(iii).: $$d_{H}( {\mathbb {R}} _{1},{\mathbb {R}} _{2})=d_{H}( {\mathbb {R}} _{2},{\mathbb {R}} _{1}),$$
(iv).: $$d_{H}( {\mathbb {R}} _{1},{\mathbb {R}} _{3})\le d_{H}( {\mathbb {R}} _{1},{\mathbb {R}} _{2})+d_{H}( {\mathbb {R}} _{2},{\mathbb {R}} _{3}).$$

The Hamming DM is defined as:

14 $$\begin{aligned} {d_{H}( {\mathbb {R}} _{1},{\mathbb {R}} _{2})=\frac{1}{2}\left[ \overset{n}{\underset{r=1}{\sum }}|\ell _{ {\mathbb {R}} _{1}}(r)-\ell _{ {\mathbb {R}} _{2}}(r)|+\frac{1}{2\pi }\overset{n}{\underset{r=1}{\sum }}|w_{ {\mathbb {R}} _{1}}(r)-w_{ {\mathbb {R}} _{2}}(r)|\right] } . \end{aligned}$$

#### Theorem 3

^40^ The function $d_{H}$ defined by Eq. (14) is a D-function of CFSs on ${\mathcal {L}}.$

### Normalized Hamming DM

#### Definition 14

^40^ The Normalized Hamming DM of CFSs is a function $d_{N}: {\mathbb {R}} ( {\mathcal {L}} )\times {\mathbb {R}} ( {\mathcal {L}} )\rightarrow [0,1]$ with the properties: for any ${\mathbb {R}} _{1}, {\mathbb {R}} _{2}, {\mathbb {R}} _{3}\in {\mathbb {R}} ( {\mathcal {L}} )$(collection of CFSs)

(i).: $$0\le d_{N}( {\mathbb {R}} _{1},{\mathbb {R}} _{2})\le 1,$$
(ii).: $$d_{N}( {\mathbb {R}} _{1},{\mathbb {R}} _{2})=0 \Longleftrightarrow {\mathbb {R}} _{1}= {\mathbb {R}} _{2},$$
(iii).: $$d_{N}( {\mathbb {R}} _{1},{\mathbb {R}} _{2})=d_{N}( {\mathbb {R}} _{2},{\mathbb {R}} _{1}),$$
(iv).: $$d_{N}( {\mathbb {R}} _{1},{\mathbb {R}} _{3})\le d_{N}( {\mathbb {R}} _{1},{\mathbb {R}} _{2})+d_{N}( {\mathbb {R}} _{2},{\mathbb {R}} _{3}).$$

The Normalized Hamming DM is defined as:

15 $$\begin{aligned} {d_{N}( {\mathbb {R}} _{1},{\mathbb {R}} _{2})=\frac{1}{2n}\left[ \overset{n}{\underset{r=1}{\sum }}|\ell _{ {\mathbb {R}} _{1}}(r)-\ell _{ {\mathbb {R}} _{2}}(r)|+\frac{1}{2\pi }\overset{n}{\underset{r=1}{\sum }}|w_{ {\mathbb {R}} _{1}}(r)-w_{ {\mathbb {R}} _{2}}(r)|\right] } . \end{aligned}$$

#### Theorem 4

^40^ The function $d_{N}$ defined by Eq. (15) is a D-function of CFSs on ${\mathcal {L}}.$

### Euclidean DM

#### Definition 15

^40^ The Euclidean DM of CFSs is a function $d_{E}: {\mathbb {R}} ( {\mathcal {L}} )\times {\mathbb {R}} ( {\mathcal {L}} )\rightarrow [0,1]$ with the properties: for any ${\mathbb {R}}_{1}, {\mathbb {R}} _{2}, {\mathbb {R}} _{3}\in {\mathbb {R}} ( {\mathcal {L}} )(collection of CFSs)$

(i).: $$0\le d_{E}( {\mathbb {R}} _{1},{\mathbb {R}} _{2})\le 1,$$
(ii).: $$d_{E}( {\mathbb {R}} _{1},{\mathbb {R}} _{2})=0 \Longleftrightarrow {\mathbb {R}} _{1}= {\mathbb {R}} _{2},$$
(iii).: $$d_{E}( {\mathbb {R}} _{1},{\mathbb {R}} _{2})=d_{E}( {\mathbb {R}} _{2},{\mathbb {R}} _{1}),$$
(iv).: $$d_{E}( {\mathbb {R}} _{1},{\mathbb {R}} _{3})\le d_{E}( {\mathbb {R}} _{1},{\mathbb {R}} _{2})+d_{E}( {\mathbb {R}} _{2},{\mathbb {R}} _{3}).$$

The Euclidean DM is defined as:

16 $$\begin{aligned} {d_{E}( {\mathbb {R}} _{1},{\mathbb {R}} _{2})=\sqrt{\frac{1}{2}\left( \overset{n}{\underset{r=1}{\sum }}\left[ \left( \ell _{ {\mathbb {R}} _{1}}(r)-\ell _{ {\mathbb {R}} _{2}}(r)\right) ^{2}+\frac{1}{2\pi ^{2}}\left( w_{ {\mathbb {R}} _{1}}(r)-w_{ {\mathbb {R}} _{2}}(r)\right) ^{2}\right] \right) }}. \end{aligned}$$

#### Theorem 5

^40^ The function $d_{E}$ defined by Eq. (16) is a D-function of CFSs on ${\mathcal {L}}.$

### Normalized Euclidean DM

#### Definition 16

^40^ The Euclidean DM of CFSs is a function $d_{\aleph }: {\mathbb {R}} ( {\mathcal {L}} )\times {\mathbb {R}} ( {\mathcal {L}} )\rightarrow [0,1]$ with the properties: for any ${\mathbb {R}} _{1}, {\mathbb {R}} _{2}, {\mathbb {R}} _{3}\in {\mathbb {R}} ( {\mathcal {L}} )($collection of CFSs)

(i).: $$0\le d_{\aleph }( {\mathbb {R}} _{1},{\mathbb {R}} _{2})\le 1,$$
(ii).: $$d_{\aleph }( {\mathbb {R}} _{1},{\mathbb {R}} _{2})=0 \Longleftrightarrow {\mathbb {R}} _{1}= {\mathbb {R}} _{2},$$
(iii).: $$d_{\aleph }( {\mathbb {R}} _{1},{\mathbb {R}} _{2})=d_{\aleph }( {\mathbb {R}} _{2},{\mathbb {R}} _{1}),$$
(iv).: $$d_{\aleph }( {\mathbb {R}} _{1},{\mathbb {R}} _{3})\le d_{\aleph }( {\mathbb {R}} _{1},{\mathbb {R}} _{2})+d_{\aleph }( {\mathbb {R}} _{2},{\mathbb {R}} _{3}).$$

The Euclidean DM is defined as:

17 $$\begin{aligned} {d_{\aleph }( {\mathbb {R}} _{1},{\mathbb {R}} _{2})=\sqrt{\frac{1}{2n}\left( \overset{n}{\underset{r=1}{\sum }}\left[ \left( \ell _{ {\mathbb {R}} _{1}}(r)-\ell _{ {\mathbb {R}} _{2}}(r)\right) ^{2}+\frac{1}{2\pi ^{2}}\left( w_{ {\mathbb {R}} _{1}}(r)-w_{ {\mathbb {R}} _{2}}(r)\right) ^{2}\right] \right) }}. \end{aligned}$$

#### Theorem 6

The function $d_{\aleph }$ defined by Eq. (17) is a D-function of CFSs on ${\mathcal {L}}.$

## Main results concerning complex fuzzy operations

In this section, some basic identities of set theory are discussed based on CF union, CF complement, CF intersection simple difference, reflection, and rotation.

### Theorem 7

Prove that for CFSs, ${\mathbb {R}} _{i};$ $$i=1,2,\ldots ,n,\ $$

8 $$\begin{aligned} {\mathbb {R}} _{1}\cup (\underset{i=1}{\overset{n}{\cap }} {\mathbb {R}} _{i})=\overset{n}{\underset{i=1}{\cap }}( {\mathbb {R}} _{1}\cup {\mathbb {R}} _{i}). \end{aligned}$$

### Proof

Let ${\mathbb {R}} _{1},$ ${\mathbb {R}} _{2},\ldots ,{\mathbb {R}} _{n}$ be *n* CFSs and $\Im _{ {\mathbb {R}} _{1}}(r)=\ell _{ {\mathbb {R}} _{1}}(r)e^{iw_{ {\mathbb {R}} _{1}}(r)},$ $\Im _{ {\mathbb {R}} _{2}}(r)=\ell _{ {\mathbb {R}} _{2}}(r)$ $e^{iw_{ {\mathbb {R}} _{2}}(r)},$ . . .,  $\Im _{ {\mathbb {R}} _{n}}(r)=\ell _{ {\mathbb {R}} _{n}}(r)e^{iw_{ {\mathbb {R}} _{n}}(r)}$ represent their membership functions, respectively. Then

9 $$\begin{aligned} {\mathbb {R}} _{1}\cup (\underset{i=1}{\overset{n}{\cap }} {\mathbb {R}} _{i})&= {\mathbb {R}} _{1}\cup \left( {\mathbb {R}} _{1}\cap {\mathbb {R}} _{2}\cap {\mathbb {R}} _{3}\cap \ldots \cap {\mathbb {R}} _{n}\right) ,\\&=\Im _{ {\mathbb {R}} _{1}}(r)\cup \left( \Im _{ {\mathbb {R}} _{1}}(r)\cap \Im _{ {\mathbb {R}} _{2}}(r)\cap \Im _{ {\mathbb {R}} _{3}}(r)\cap \ldots \cap \Im _{ {\mathbb {R}} _{n}}(r)\right) , =\max \left\{ \ell _{ {\mathbb {R}} _{1}}(r),\min \left( \ell _{ {\mathbb {R}} _{1}}(r),\ell _{ {\mathbb {R}} _{2}}(r),\ldots ,\ell _{ {\mathbb {R}} _{n}}(r)\right) \right\} e^{i\max \left\{ w_{ {\mathbb {R}} _{1}}(r),\min \left( w_{ {\mathbb {R}} _{1}}(r),w_{ {\mathbb {R}} _{2}}(r),\ldots ,w_{ {\mathbb {R}} _{n}}(r)\right) \right\} }. \end{aligned}$$

Also,

10 $$\begin{aligned} \overset{n}{\underset{i=1}{\cap }}( {\mathbb {R}} _{1}\cup {\mathbb {R}} _{i})&=\left( {\mathbb {R}} _{1}\cup {\mathbb {R}} _{1}\right) \cap \left( {\mathbb {R}} _{1}\cup {\mathbb {R}} _{2}\right) \cap \ldots \cap \left( {\mathbb {R}} _{1}\cup {\mathbb {R}} _{n}\right) ,\\&=\left( \Im _{ {\mathbb {R}} _{1}}(r)\cup \Im _{ {\mathbb {R}} _{1}}(r)\right) \cap \left( \Im _{ {\mathbb {R}} _{1}}(r)\cup \Im _{ {\mathbb {R}} _{2}}(r)\right) \cap \ldots \cap \left( \Im _{ {\mathbb {R}} _{1}}(r)\cup \Im _{ {\mathbb {R}} _{n}}(r)\right) ,\\&=\min \left\{ \begin{array}{c} \max \left( \ell _{ {\mathbb {R}} _{1}}(r),\ell _{ {\mathbb {R}} _{1}}(r)\right) ,\max \left( \ell _{ {\mathbb {R}} _{1}}(r),\ell _{ {\mathbb {R}} _{2}}(r)\right) ,\ldots , \max \left( \ell _{ {\mathbb {R}} _{1}}(r),\ell _{ {\mathbb {R}} _{n}}(r)\right) \end{array} \right\} \\&\quad e^{i\min \left\{ \begin{array}{c} \max \left( w_{ {\mathbb {R}} _{1}}(r),w_{ {\mathbb {R}} _{1}}(r)\right) ,\max \left( w_{ {\mathbb {R}} _{1}}(r),w_{ {\mathbb {R}} _{2}}(r)\right) ,\ldots , \max \left( w_{ {\mathbb {R}} _{1}}(r),w_{ {\mathbb {R}} _{n}}(r)\right) \end{array} \right\} .} \end{aligned}$$

To prove the generalized form of De-Margon law, we take some cases.

Case 1. If $\ell _{ {\mathbb {R}} _{1}}(r)\le \ell _{ {\mathbb {R}} _{2}}(r)\le \cdots \le \ell _{ {\mathbb {R}} _{n}}(r)$ and $w_{ {\mathbb {R}} _{1}}(r)\le w_{ {\mathbb {R}} _{2}}(r)\le \cdots \le w_{ {\mathbb {R}} _{n}}(r)$ then (20) and (21) implies that

11 $$\begin{aligned} {\mathbb {R}} _{1}\cup (\underset{i=1}{\overset{n}{\cap }} {\mathbb {R}} _{i})&=\max \left\{ \ell _{ {\mathbb {R}} _{1}}(r),\min \left( \ell _{ {\mathbb {R}} _{1}}(r),\ell _{ {\mathbb {R}} _{2}}(r),\ldots ,\ell _{ {\mathbb {R}} _{n}}(r)\right) \right\} e^{i\max \left\{ w_{ {\mathbb {R}} _{1}}(r),\min \left( w_{ {\mathbb {R}} _{1}}(r),w_{ {\mathbb {R}} _{2}}(r),\ldots ,w_{ {\mathbb {R}} _{n}}(r)\right) \right\} },\\&=\max \left\{ \ell _{ {\mathbb {R}} _{1}}(r),\ell _{ {\mathbb {R}} _{1}}(r)\right\} e^{i\max \left\{ w_{ {\mathbb {R}} _{1}}(r),w_{ {\mathbb {R}} _{1}}(r)\right\} },\\&=\ell _{ {\mathbb {R}} _{1}}(r)e^{iw_{ {\mathbb {R}} _{1}}(r)}. \end{aligned}$$

Also,

12 $$\begin{aligned} \overset{n}{\underset{i=1}{\cap }}( {\mathbb {R}} _{1}\cup {\mathbb {R}} _{i})&=\min \left\{ \begin{array}{c} \max \left( \ell _{ {\mathbb {R}} _{1}}(r),\ell _{ {\mathbb {R}} _{1}}(r)\right) ,\max \left( \ell _{ {\mathbb {R}} _{1}}(r),\ell _{ {\mathbb {R}} _{2}}(r)\right) ,\ldots , \max \left( \ell _{ {\mathbb {R}} _{1}}(r),\ell _{ {\mathbb {R}} _{n}}(r)\right) \end{array} \right\} \\&\quad e^{i\min \left\{ \begin{array}{c} \max \left( w_{ {\mathbb {R}} _{1}}(r),w_{ {\mathbb {R}} _{1}}(r)\right) ,\max \left( w_{ {\mathbb {R}} _{1}}(r),w_{ {\mathbb {R}} _{2}}(r)\right) ,\ldots , \max \left( w_{ {\mathbb {R}} _{1}}(r),w_{ {\mathbb {R}} _{n}}(r)\right) \end{array} \right\} .},\\&=\min \left\{ \ell _{ {\mathbb {R}} _{1}}(r),\ell _{ {\mathbb {R}} _{2}}(r),\ell _{ {\mathbb {R}} _{3}}(r),\ldots ,\ell _{ {\mathbb {R}} _{n}}(r)\right\} e^{i\min \left\{ w_{ {\mathbb {R}} _{1}}(r),w_{ {\mathbb {R}} _{2}}(r),\ldots ,w_{ {\mathbb {R}} _{n}}(r)\right\} },\\&=\ell _{ {\mathbb {R}} _{1}}(r)e^{iw_{ {\mathbb {R}} _{1}}(r)}. \end{aligned}$$

From (11) and (12), we have

$$\begin{aligned} {\mathbb {R}} _{1}\cup (\underset{i=1}{\overset{n}{\cap }} {\mathbb {R}} _{i})=\overset{n}{\underset{i=1}{\cap }}( {\mathbb {R}} _{1}\cup {\mathbb {R}} _{i}). \end{aligned}$$

Case 2. If $\ell _{ {\mathbb {R}} _{n}}(r)\le \ell _{ {\mathbb {R}} _{n-1}}(r)\le \cdots \le \ell _{ {\mathbb {R}} _{1}}(r)$ and $w_{ {\mathbb {R}} _{n}}(r)\le w_{ {\mathbb {R}} _{n-1}}(r)\le \cdots \le w_{ {\mathbb {R}} _{1}}(r)$ then (20) and (21) implies that

13 $$\begin{aligned} {\mathbb {R}} _{1}\cup (\underset{i=1}{\overset{n}{\cap }} {\mathbb {R}} _{i})&=\max \left\{ \ell _{ {\mathbb {R}} _{1}}(r),\min \left( \ell _{ {\mathbb {R}} _{1}}(r),\ell _{ {\mathbb {R}} _{2}}(r),\ldots ,\ell _{ {\mathbb {R}} _{n}}(r)\right) \right\} e^{i\max \left\{ w_{ {\mathbb {R}} _{1}}(r),\min \left( w_{ {\mathbb {R}} _{1}}(r),w_{ {\mathbb {R}} _{2}}(r),\ldots ,w_{ {\mathbb {R}} _{n}}(r)\right) \right\} },\\&=\max \left\{ \ell _{ {\mathbb {R}} _{1}}(r),\ell _{ {\mathbb {R}} _{n}}(r)\right\} e^{i\max \left\{ w_{ {\mathbb {R}} _{1}}(r),w_{ {\mathbb {R}} _{n}}(r)\right\} },\\&=\ell _{ {\mathbb {R}} _{1}}(r)e^{iw_{ {\mathbb {R}} _{1}}(r)}. \end{aligned}$$

Also,

14 $$\begin{aligned} \overset{n}{\underset{i=1}{\cap }}( {\mathbb {R}} _{1}\cup {\mathbb {R}} _{i})&=\min \left\{ \begin{array}{c} \max \left( \ell _{ {\mathbb {R}} _{1}}(r),\ell _{ {\mathbb {R}} _{1}}(r)\right) ,\max \left( \ell _{ {\mathbb {R}} _{1}}(r),\ell _{ {\mathbb {R}} _{2}}(r)\right) ,\ldots , \max \left( \ell _{ {\mathbb {R}} _{1}}(r),\ell _{ {\mathbb {R}} _{n}}(r)\right) \end{array} \right\} \\&\quad e^{i\min \left\{ \begin{array}{c} \max \left( w_{ {\mathbb {R}} _{1}}(r),w_{ {\mathbb {R}} _{1}}(r)\right) ,\max \left( w_{ {\mathbb {R}} _{1}}(r),w_{ {\mathbb {R}} _{2}}(r)\right) ,\ldots , \max \left( w_{ {\mathbb {R}} _{1}}(r),w_{ {\mathbb {R}} _{n}}(r)\right) \end{array} \right\} .},\\&=\min \left\{ \ell _{ {\mathbb {R}} _{1}}(r),\ell _{ {\mathbb {R}} _{1}}(r),\ell _{ {\mathbb {R}} _{1}}(r),\ldots ,\ell _{ {\mathbb {R}} _{1}}(r)\right\} e^{i\min \left\{ w_{ {\mathbb {R}} _{1}}(r),w_{ {\mathbb {R}} _{1}}(r),\ldots ,w_{ {\mathbb {R}} _{1}}(r)\right\} .},\\&=\ell _{ {\mathbb {R}} _{1}}(r)e^{iw_{ {\mathbb {R}} _{1}}(r)}. \end{aligned}$$

From (13) and (14), we have

$$\begin{aligned} {\mathbb {R}} _{1}\cup (\underset{i=1}{\overset{n}{\cap }} {\mathbb {R}} _{i})=\overset{n}{\underset{i=1}{\cap }}( {\mathbb {R}} _{1}\cup {\mathbb {R}} _{i}). \end{aligned}$$

The Proof is similar for other cases. $\square$

### Theorem 8

Prove that for CFSs, ${\mathbb {R}} _{i};$ $$i=1,2,\ldots ,n\ $$

15 $$\begin{aligned} {\mathbb {R}} _{1}\cap (\underset{i=1}{\overset{n}{\cup }} {\mathbb {R}} _{i})=\overset{n}{\underset{i=1}{\cup }}( {\mathbb {R}} _{1}\cap {\mathbb {R}} _{i}). \end{aligned}$$

### Proof

Let ${\mathbb {R}} _{1},$ ${\mathbb {R}} _{2},\ldots ,{\mathbb {R}} _{n}$ be *n* CFSs and $\Im _{ {\mathbb {R}} _{1}}(r)=\ell _{ {\mathbb {R}} _{1}}(r)e^{iw_{ {\mathbb {R}} _{1}}(r)},$ $\Im _{ {\mathbb {R}} _{2}}(r)=$ $\ell _{ {\mathbb {R}} _{2}}(r)$ $e^{iw_{ {\mathbb {R}} _{2}}(r)},$ . . .,  $\Im _{ {\mathbb {R}} _{n}}(r)=\ell _{ {\mathbb {R}} _{n}}(r)e^{iw_{ {\mathbb {R}} _{n}}(r)}$ represent their membership functions, respectively. Then

16 $$\begin{aligned} {\mathbb {R}} _{1}\cap (\underset{i=1}{\overset{n}{\cup }} {\mathbb {R}} _{i})&= {\mathbb {R}} _{1}\cap \left( {\mathbb {R}} _{1}\cup {\mathbb {R}} _{2}\cup {\mathbb {R}} _{3}\cup \ldots \cup {\mathbb {R}} _{n}\right) ,\\&=\Im _{ {\mathbb {R}} _{1}}(r)\cap \left( \Im _{ {\mathbb {R}} _{1}}(r)\cup \Im _{ {\mathbb {R}} _{2}}(r)\cup \Im _{ {\mathbb {R}} _{3}}(r)\cup \ldots \cup \Im _{ {\mathbb {R}} _{n}}(r)\right) ,\\&=\min \left\{ \ell _{ {\mathbb {R}} _{1}}(r),\max \left( \ell _{ {\mathbb {R}} _{1}}(r),\ell _{ {\mathbb {R}} _{2}}(r),\ldots ,\ell _{ {\mathbb {R}} _{n}}(r)\right) \right\} e^{i\min \left\{ w_{ {\mathbb {R}} _{1}}(r),\max \left( w_{ {\mathbb {R}} _{1}}(r),w_{ {\mathbb {R}} _{2}}(r),\ldots ,w_{ {\mathbb {R}} _{n}}(r)\right) \right\} }, \end{aligned}$$

Also,

17 $$\begin{aligned} \overset{n}{\underset{i=1}{\cup }}( {\mathbb {R}} _{1}\cap {\mathbb {R}} _{i})&=\left( {\mathbb {R}} _{1}\cap {\mathbb {R}} _{1}\right) \cup \left( {\mathbb {R}} _{1}\cap {\mathbb {R}} _{2}\right) \cup \ldots \cup \left( {\mathbb {R}} _{1}\cap {\mathbb {R}} _{n}\right) ,\\&=\left( \Im _{ {\mathbb {R}} _{1}}(r)\cap \Im _{ {\mathbb {R}} _{1}}(r)\right) \cup \left( \Im _{ {\mathbb {R}} _{1}}(r)\cap \Im _{ {\mathbb {R}} _{2}}(r)\right) \cup \ldots \cup \left( \Im _{ {\mathbb {R}} _{1}}(r)\cap \Im _{ {\mathbb {R}} _{n}}(r)\right) ,\\&=\max \left\{ \begin{array}{c} \min \left( \ell _{ {\mathbb {R}} _{1}}(r),\ell _{ {\mathbb {R}} _{1}}(r)\right) ,\min \left( \ell _{ {\mathbb {R}} _{1}}(r),\ell _{ {\mathbb {R}} _{2}}(r)\right) ,\ldots , \min \left( \ell _{ {\mathbb {R}} _{1}}(r),\ell _{ {\mathbb {R}} _{n}}(r)\right) \end{array} \right\} \\&\quad e^{i\max \left\{ \begin{array}{c} \min \left( w_{ {\mathbb {R}} _{1}}(r),w_{ {\mathbb {R}} _{1}}(r)\right) ,\min \left( w_{ {\mathbb {R}} _{1}}(r),w_{ {\mathbb {R}} _{2}}(r)\right) ,\ldots , \min \left( w_{ {\mathbb {R}} _{1}}(r),w_{ {\mathbb {R}} _{n}}(r)\right) \end{array} \right\} .} \end{aligned}$$

To prove the generalized form of De-Margon law, we take some cases.

Case 1. If $\ell _{ {\mathbb {R}} _{1}}(r)\le \ell _{ {\mathbb {R}} _{2}}(r)\le \cdots \le \ell _{ {\mathbb {R}} _{n}}(r)$ and $w_{ {\mathbb {R}} _{1}}(r)\le w_{ {\mathbb {R}} _{2}}(r)\le \ldots \le w_{ {\mathbb {R}} _{n}}(r)$ then (20) and (21) implies that

18 $$\begin{aligned} {\mathbb {R}} _{1}\cap (\underset{i=1}{\overset{n}{\cup }} {\mathbb {R}} _{i})&=\min \left\{ \ell _{ {\mathbb {R}} _{1}}(r),\max \left( \ell _{ {\mathbb {R}} _{1}}(r),\ell _{ {\mathbb {R}} _{2}}(r),\ldots ,\ell _{ {\mathbb {R}} _{n}}(r)\right) \right\} e^{i\min \left\{ w_{ {\mathbb {R}} _{1}}(r),\max \left( w_{ {\mathbb {R}} _{1}}(r),w_{ {\mathbb {R}} _{2}}(r),\ldots ,w_{ {\mathbb {R}} _{n}}(r)\right) \right\} },\\&=\min \left\{ \ell _{ {\mathbb {R}} _{1}}(r),\ell _{ {\mathbb {R}} _{n}}(r)\right\} e^{i\min \left\{ w_{ {\mathbb {R}} _{1}}(r),w_{ {\mathbb {R}} _{1}}(r)\right\} },\\&=\ell _{ {\mathbb {R}} _{1}}(r)e^{iw_{ {\mathbb {R}} _{1}}(r)}. \end{aligned}$$

Also,

19 $$\begin{aligned} \overset{n}{\underset{i=1}{\cup }}( {\mathbb {R}} _{1}\cap {\mathbb {R}} _{i})&=\max \left\{ \begin{array}{c} \min \left( \ell _{ {\mathbb {R}} _{1}}(r),\ell _{ {\mathbb {R}} _{1}}(r)\right) ,\min \left( \ell _{ {\mathbb {R}} _{1}}(r),\ell _{ {\mathbb {R}} _{2}}(r)\right) ,\ldots , \min \left( \ell _{ {\mathbb {R}} _{1}}(r),\ell _{ {\mathbb {R}} _{n}}(r)\right) \end{array} \right\} \\&\quad e^{i\max \left\{ \begin{array}{c} \min \left( w_{ {\mathbb {R}} _{1}}(r),w_{ {\mathbb {R}} _{1}}(r)\right) ,\min \left( w_{ {\mathbb {R}} _{1}}(r),w_{ {\mathbb {R}} _{2}}(r)\right) ,\ldots , \min \left( w_{ {\mathbb {R}} _{1}}(r),w_{ {\mathbb {R}} _{n}}(r)\right) \end{array} \right\} .},\\&=\max \left\{ \ell _{ {\mathbb {R}} _{1}}(r),\ell _{ {\mathbb {R}} _{1}}(r),\ell _{ {\mathbb {R}} _{1}}(r),\ldots ,\ell _{ {\mathbb {R}} _{1}}(r)\right\} e^{i\min \left\{ w_{ {\mathbb {R}} _{1}}(r),w_{ {\mathbb {R}} _{1}}(r),\ldots ,w_{ {\mathbb {R}} _{1}}(r)\right\} .},\\&=\ell _{ {\mathbb {R}} _{1}}(r)e^{iw_{ {\mathbb {R}} _{1}}(r)}. \end{aligned}$$

From (18) and (19), we have

$$\begin{aligned} {\mathbb {R}} _{1}\cup (\underset{i=1}{\overset{n}{\cap }} {\mathbb {R}} _{i})=\overset{n}{\underset{i=1}{\cap }}( {\mathbb {R}} _{1}\cup {\mathbb {R}} _{i}). \end{aligned}$$

Case 2. If $\ell _{ {\mathbb {R}} _{n}}(r)\le \ell _{ {\mathbb {R}} _{n-1}}(r)\le \cdots \le \ell _{ {\mathbb {R}} _{1}}(r)$ and $w_{ {\mathbb {R}} _{n}}(r)\le w_{ {\mathbb {R}} _{n-1}}(r)\le \cdots \le w_{ {\mathbb {R}} _{1}}(r)$ then (20) and (21) implies that

20 $$\begin{aligned} {\mathbb {R}} _{1}\cap (\underset{i=1}{\overset{n}{\cup }} {\mathbb {R}} _{i})&=\min \left\{ \ell _{ {\mathbb {R}} _{1}}(r),\max \left( \ell _{ {\mathbb {R}} _{1}}(r),\ell _{ {\mathbb {R}} _{2}}(r),\ldots ,\ell _{ {\mathbb {R}} _{n}}(r)\right) \right\} e^{i\min \left\{ w_{ {\mathbb {R}} _{1}}(r),\max \left( w_{ {\mathbb {R}} _{1}}(r),w_{ {\mathbb {R}} _{2}}(r),\ldots ,w_{ {\mathbb {R}} _{n}}(r)\right) \right\} },\\&=\min \left\{ \ell _{ {\mathbb {R}} _{1}}(r),\ell _{ {\mathbb {R}} _{1}}(r)\right\} e^{i\max \left\{ w_{ {\mathbb {R}} _{1}}(r),w_{ {\mathbb {R}} _{1}}(r)\right\} },\\&=\ell _{ {\mathbb {R}} _{1}}(r)e^{iw_{ {\mathbb {R}} _{1}}(r)}. \end{aligned}$$

Also,

21 $$\begin{aligned} \overset{n}{\underset{i=1}{\cup }}( {\mathbb {R}} _{1}\cap {\mathbb {R}} _{i})&=\max \left\{ \begin{array}{c} \min \left( \ell _{ {\mathbb {R}} _{1}}(r),\ell _{ {\mathbb {R}} _{1}}(r)\right) ,\min \left( \ell _{ {\mathbb {R}} _{1}}(r),\ell _{ {\mathbb {R}} _{2}}(r)\right) ,\ldots , \min \left( \ell _{ {\mathbb {R}} _{1}}(r),\ell _{ {\mathbb {R}} _{n}}(r)\right) \end{array} \right\} \\&\quad e^{i\max \left\{ \begin{array}{c} \min \left( w_{ {\mathbb {R}} _{1}}(r),w_{ {\mathbb {R}} _{1}}(r)\right) ,\min \left( w_{ {\mathbb {R}} _{1}}(r),w_{ {\mathbb {R}} _{2}}(r)\right) ,\ldots , \min \left( w_{ {\mathbb {R}} _{1}}(r),w_{ {\mathbb {R}} _{n}}(r)\right) \end{array} \right\} .},\\&=\max \left\{ \ell _{ {\mathbb {R}} _{1}}(r),\ell _{ {\mathbb {R}} _{2}}(r),\ell _{ {\mathbb {R}} _{3}}(r),\ldots ,\ell _{ {\mathbb {R}} _{n}}(r)\right\} e^{i\max \left\{ w_{ {\mathbb {R}} _{1}}(r),w_{ {\mathbb {R}} _{2}}(r),\ldots ,w_{ {\mathbb {R}} _{n}}(r)\right\} .},\\&=\ell _{ {\mathbb {R}} _{1}}(r)e^{iw_{ {\mathbb {R}} _{1}}(r)}. \end{aligned}$$

From (20) and (21), we have

$$\begin{aligned} {\mathbb {R}} _{1}\cup (\underset{i=1}{\overset{n}{\cap }} {\mathbb {R}} _{i})=\overset{n}{\underset{i=1}{\cap }}( {\mathbb {R}} _{1}\cup {\mathbb {R}} _{i}). \end{aligned}$$

The Proof is similar for other cases. $\square$

### Theorem 9

Let ${\mathbb {R}} _{1},{\mathbb {R}} _{2}$ and ${\mathbb {R}} _{3}$ be three complex fuzzy sets on U.then, complex fuzzy standard union, standard intersection, complement, and bounded difference satisfy the the following result.

22 $$\begin{aligned} \left( {\mathbb {R}} _{1}\cap {\mathbb {R}} _{2}^{c}\right) \cup \left( {\mathbb {R}} _{2}\cap {\mathbb {R}} _{3}^{c}\right) =\left( {\mathbb {R}} _{1}\cup {\mathbb {R}} _{2}\right) -( {\mathbb {R}} _{2}\cap {\mathbb {R}} _{3}). \end{aligned}$$

### Proof

Let $\Im _{ {\mathbb {R}} _{1}}(r)=\ell _{ {\mathbb {R}} _{1}}(r)e^{i\lambda _{ {\mathbb {R}} _{1}}(r)},\Im _{ {\mathbb {R}} _{2}}(r)=\ell _{ {\mathbb {R}} _{2}}(r)e^{i\lambda _{ {\mathbb {R}} _{2}}(r)}$and $\Im _{ {\mathbb {R}} _{3}}(r)=\ell _{ {\mathbb {R}} _{3}}(r)e^{i\lambda _{ {\mathbb {R}} _{3}}(r)}$ are the membership functions of ${\mathbb {R}} _{1},{\mathbb {R}} _{2}$ and ${\mathbb {R}} _{3},$ respectively. Taking left-hand side of Eq. (22),

23 $$\begin{aligned} \left( {\mathbb {R}} _{1}\cap {\mathbb {R}} _{2}^{c}\right) \cup \left( {\mathbb {R}} _{2}\cap {\mathbb {R}} _{3}^{c}\right)&=\left[ \Im _{ {\mathbb {R}} _{1}}(r)\cap \Im _{ {\mathbb {R}} _{2}^{c}}(r)\right] \cup \left[ \Im _{ {\mathbb {R}} _{2}}(r)\cap \Im _{ {\mathbb {R}} _{3}^{c}}(r)\right] ,\\&=\left[ \ell _{ {\mathbb {R}} _{1}}(r)e^{i\lambda _{ {\mathbb {R}} _{1}}(r)}\cap (1-\ell _{ {\mathbb {R}} _{2}}(r))e^{i\lambda _{ {\mathbb {R}} _{2}}(r)}\right] \cup \\&\quad \left[ \ell _{ {\mathbb {R}} _{2}}(r)e^{i\lambda _{ {\mathbb {R}} _{2}}(r)}\cap (1-\ell _{ {\mathbb {R}} _{3}}(r))e^{i\lambda _{ {\mathbb {R}} _{3}}(r)}\right] ,\\&=\left[ \min (\ell _{ {\mathbb {R}} _{1}}(r),1-\ell _{ {\mathbb {R}} _{2}}(r))e^{i\min (w_{ {\mathbb {R}} _{1}}(r),w_{ {\mathbb {R}} _{2}}(r)}\right] \cup \\&\quad \left[ \min (\ell _{ {\mathbb {R}} _{2}}(r),1-\ell _{ {\mathbb {R}} _{3}}(r))e^{i\min (w_{ {\mathbb {R}} _{2}}(r),w_{ {\mathbb {R}} _{3}}(r)}\right] ,\\&=\max \left\{ \begin{array}{c} \min (\ell _{ {\mathbb {R}} _{1}}(r),1-\ell _{ {\mathbb {R}} _{2}}(r))e^{i\min (w_{ {\mathbb {R}} _{1}}(r),w_{ {\mathbb {R}} _{2}}(r))},\\ \min (\ell _{ {\mathbb {R}} _{2}}(r),1-\ell _{ {\mathbb {R}} _{3}}(r))e^{i\min (w_{ {\mathbb {R}} _{2}}(r),w_{ {\mathbb {R}} _{3}}(r))} \end{array} \right\} ,\\&=\max \left\{ \min (\ell _{ {\mathbb {R}} _{1}}(r),1-\ell _{ {\mathbb {R}} _{2}}(r)),\min (\ell _{ {\mathbb {R}} _{2}}(r),1-\ell _{ {\mathbb {R}} _{3}}(r))\right\} \\&\quad e^{i\max \left( \min (w_{ {\mathbb {R}} _{1}}(r),w_{ {\mathbb {R}} _{2}}(r)),\min (w_{ {\mathbb {R}} _{2}}(r),w_{ {\mathbb {R}} _{3}}(r))\right) }. \end{aligned}$$

Now taking right-hand side of Eq. (22),

24 $$\begin{aligned} \left( {\mathbb {R}} _{1}\cup {\mathbb {R}} _{2}\right) \cap \left( {\mathbb {R}} _{2}\cap {\mathbb {R}} _{3}\right) ^{c}&=\left( {\mathbb {R}} _{1}\cup {\mathbb {R}} _{2}\right) \cap \left( {\mathbb {R}} _{2}^{c}\cup {\mathbb {R}} _{3}^{c}\right) \\&=\left[ \Im _{ {\mathbb {R}} _{1}}(r)\cup \Im _{ {\mathbb {R}} _{2}}(r)\right] \cap \left[ \Im _{ {\mathbb {R}} _{2}^{c}}(r)\cup \Im _{ {\mathbb {R}} _{3}^{c}}(r)\right] ,\\&=\left[ \ell _{ {\mathbb {R}} _{1}}(r)e^{iw_{ {\mathbb {R}} _{1}}(r)}\cup \ell _{ {\mathbb {R}} _{2}}(r)e^{iw_{ {\mathbb {R}} _{2}}(r)}\right] \cap \\&\quad \left[ \max \left( 1-\ell _{ {\mathbb {R}} _{2}}(r),1-\ell _{ {\mathbb {R}} _{3}}(r)\right) e^{i\max (w_{ {\mathbb {R}} _{2}}(r),w_{ {\mathbb {R}} _{3}}(r))}\right] ,\\&=\min \left\{ \begin{array}{c} \max (\ell _{ {\mathbb {R}} _{1}}(r),\ell _{ {\mathbb {R}} _{2}}(r))e^{i\max (w_{ {\mathbb {R}} _{1}}(r),w_{ {\mathbb {R}} _{2}}(r))},\\ \max \left( 1-\ell _{ {\mathbb {R}} _{2}}(r),1-\ell _{ {\mathbb {R}} _{3}}(r)\right) e^{i\max (w_{ {\mathbb {R}} _{2}}(r),w_{ {\mathbb {R}} _{3}}(r))} \end{array} \right\} \\&=\min \left\{ \max (\ell _{ {\mathbb {R}} _{1}}(r),\ell _{ {\mathbb {R}} _{2}}(r)),\max \left( 1-\ell _{ {\mathbb {R}} _{2}}(r),1-\ell _{ {\mathbb {R}} _{3}}(r)\right) \right\} \\&\quad e^{i\min \left( \max (w_{ {\mathbb {R}} _{1}}(r),w_{ {\mathbb {R}} _{2}}(r)),\max (w_{ {\mathbb {R}} _{2}}(r),w_{ {\mathbb {R}} _{3}}(r))\right) }. \end{aligned}$$

Now, to prove the result here, we discuss a few cases: $\square$

Case 1. If $\ell _{ {\mathbb {R}} _{1}}(r)\le \ell _{ {\mathbb {R}} _{2}}(r)\le \ell _{ {\mathbb {R}} _{3}}(r)$ and $w_{ {\mathbb {R}} _{1}}(r)\le w_{ {\mathbb {R}} _{2}}(r)\le w_{ {\mathbb {R}} _{3}}(r)$ then (23) and (24) implies that

25 $$\begin{aligned} \left( {\mathbb {R}} _{1}\cap {\mathbb {R}} _{2}^{c}\right) \cup \left( {\mathbb {R}} _{2}\cap {\mathbb {R}} _{3}^{c}\right)&=\max \left\{ \min (\ell _{ {\mathbb {R}} _{1}}(r),1-\ell _{ {\mathbb {R}} _{2}}(r)),\min (\ell _{ {\mathbb {R}} _{2}}(r),1-\ell _{ {\mathbb {R}} _{3}}(r))\right\} \\&\quad e^{i\max \left( \min (w_{ {\mathbb {R}} _{1}}(r),w_{ {\mathbb {R}} _{2}}(r)),\min (w_{ {\mathbb {R}} _{2}}(r),w_{ {\mathbb {R}} _{3}}(r))\right) },\\&=\max \left\{ 1-\ell _{ {\mathbb {R}} _{2}}(r),1-\ell _{ {\mathbb {R}} _{3}}(r)\right\} e^{i\max \left( w_{ {\mathbb {R}} _{1}}(r),w_{ {\mathbb {R}} _{2}}(r)\right) }\\&=\left( 1-\ell _{ {\mathbb {R}} _{2}}(r)\right) e^{iw_{ {\mathbb {R}} _{2}}(r)}. \end{aligned}$$

26 $$\begin{aligned} \left( {\mathbb {R}} _{1}\cup {\mathbb {R}} _{2}\right) \cap \left( {\mathbb {R}} _{2}\cap {\mathbb {R}} _{3}\right) ^{c}&=\min \left\{ \max (\ell _{ {\mathbb {R}} _{1}}(r),\ell _{ {\mathbb {R}} _{2}}(r)),\max \left( 1-\ell _{ {\mathbb {R}} _{2}}(r),1-\ell _{ {\mathbb {R}} _{3}}(r)\right) \right\} \\&\quad e^{i\min \left( \max (w_{ {\mathbb {R}} _{1}}(r),w_{ {\mathbb {R}} _{2}}(r)),\max (w_{ {\mathbb {R}} _{2}}(r),w_{ {\mathbb {R}} _{3}}(r))\right) }\\&=\min \left\{ \ell _{ {\mathbb {R}} _{2}}(r),1-\ell _{ {\mathbb {R}} _{2}}(r)\right\} e^{i\min \left( w_{ {\mathbb {R}} _{2}}(r)),w_{ {\mathbb {R}} _{3}}(r)\right) }\\&=1-\ell _{ {\mathbb {R}} _{2}}(r)e^{iw_{ {\mathbb {R}} _{2}}(r)}. \end{aligned}$$

From (25) and (26), we have

$$\begin{aligned} \left( {\mathbb {R}} _{1}\cap {\mathbb {R}} _{2}^{c}\right) \cup \left( {\mathbb {R}} _{2}\cap {\mathbb {R}} _{3}^{c}\right) =\left( {\mathbb {R}} _{1}\cup {\mathbb {R}} _{2}\right) -( {\mathbb {R}} _{2}\cap {\mathbb {R}} _{3}). \end{aligned}$$

Case 2. If $\ell _{ {\mathbb {R}} _{3}}(r)\le \ell _{ {\mathbb {R}} _{2}}(r)\le \ell _{ {\mathbb {R}} _{1}}(r)$ and $w_{ {\mathbb {R}} _{3}}(r)\le w_{ {\mathbb {R}} _{2}}(r)\le w_{ {\mathbb {R}} _{1}}(r)$ then (23) and (24) implies that

27 $$\begin{aligned} \left( {\mathbb {R}} _{1}\cap {\mathbb {R}} _{2}^{c}\right) \cup \left( {\mathbb {R}} _{2}\cap {\mathbb {R}} _{3}^{c}\right)&=\max \left\{ \min (\ell _{ {\mathbb {R}} _{1}}(r),1-\ell _{ {\mathbb {R}} _{2}}(r)),\min (\ell _{ {\mathbb {R}} _{2}}(r),1-\ell _{ {\mathbb {R}} _{3}}(r))\right\} \nonumber \\&\quad e^{i\max \left( \min (w_{ {\mathbb {R}} _{1}}(r),w_{ {\mathbb {R}} _{2}}(r)),\min (w_{ {\mathbb {R}} _{2}}(r),w_{ {\mathbb {R}} _{3}}(r))\right) },\nonumber \\&=\max \left\{ \ell _{ {\mathbb {R}} _{1}}(r),\ell _{ {\mathbb {R}} _{2}}(r)\right\} e^{i\max \left( w_{ {\mathbb {R}} _{2}}(r),w_{ {\mathbb {R}} _{3}}(r)\right) }\nonumber \\&=\ell _{ {\mathbb {R}} _{1}}(r)e^{iw_{ {\mathbb {R}} _{2}}(r)}, \end{aligned}$$

28 $$\begin{aligned} \left( {\mathbb {R}} _{1}\cup {\mathbb {R}} _{2}\right) \cap \left( {\mathbb {R}} _{2}\cap {\mathbb {R}} _{3}\right) ^{c}&=\min \left\{ \max (\ell _{ {\mathbb {R}} _{1}}(r),\ell _{ {\mathbb {R}} _{2}}(r)),\max \left( 1-\ell _{ {\mathbb {R}} _{2}}(r),1-\ell _{ {\mathbb {R}} _{3}}(r)\right) \right\} \nonumber \\&\quad e^{i\min \left( \max (w_{ {\mathbb {R}} _{1}}(r),w_{ {\mathbb {R}} _{2}}(r)),\max (w_{ {\mathbb {R}} _{2}}(r),w_{ {\mathbb {R}} _{3}}(r))\right) }\nonumber \\&=\min \left\{ \ell _{ {\mathbb {R}} _{1}}(r),1-\ell _{ {\mathbb {R}} _{3}}(r)\right\} e^{i\min \left( w_{ {\mathbb {R}} _{1}}(r)),w_{ {\mathbb {R}} _{2}}(r)\right) }\nonumber \\&=\ell _{ {\mathbb {R}} _{1}}(r)e^{iw_{ {\mathbb {R}} _{2}}(r)}. \end{aligned}$$

From (27) and (28), we have

$$\begin{aligned} \left( {\mathbb {R}} _{1}\cap {\mathbb {R}} _{2}^{c}\right) \cup \left( {\mathbb {R}} _{2}\cap {\mathbb {R}} _{3}^{c}\right) =\left( {\mathbb {R}} _{1}\cup {\mathbb {R}} _{2}\right) -( {\mathbb {R}} _{2}\cap {\mathbb {R}} _{3}). \end{aligned}$$

The Proof is similar for other cases.

### Theorem 10

For $$ {\mathbb {R}} _{1}\ $$and ${\mathbb {R}} _{2}$ the CFSs on the universal U then CF standard union,standard intersection and complement must satisfy the following result:

29 $$\begin{aligned} \left( {\mathbb {R}} _{1}^{c}\cup {\mathbb {R}} _{2}^{c}\right) \cap {\mathbb {R}} _{1}^{c}= {\mathbb {R}} _{1}^{c}. \end{aligned}$$

### Proof

Let $\Im _{ {\mathbb {R}} _{1}}(r)=\ell _{ {\mathbb {R}} _{1}}(r)e^{iw_{ {\mathbb {R}} _{1}}(r)},$ $\Im _{ {\mathbb {R}} _{2}}(r)=\ell _{ {\mathbb {R}} _{2}}(r)e^{iw_{ {\mathbb {R}} _{2}}(r)}$ and $\Im _{ {\mathbb {R}} _{3}}(r)=\ell _{ {\mathbb {R}} _{3}}(r)e^{iw_{ {\mathbb {R}} _{3}}(r)}$ are the membership functions of $$ {\mathbb {R}} _{1}\ $$and ${\mathbb {R}} _{2},$ respectively. Then

$$\begin{aligned} \left( {\mathbb {R}} _{1}^{c}\cup {\mathbb {R}} _{2}^{c}\right)&=\Im _{ {\mathbb {R}} _{1}^{c}}(r)\cup \Im _{ {\mathbb {R}} _{2}^{c}}(r)=\ell _{ {\mathbb {R}} _{1}}^{c}(r)e^{iw_{ {\mathbb {R}} _{1}}(r)}\cup \ell _{ {\mathbb {R}} _{2}}^{c}(r)e^{iw_{ {\mathbb {R}} _{2}}(r)}\\&=\max (1-\ell _{ {\mathbb {R}} _{1}}(r),1-\ell _{ {\mathbb {R}} _{2}}(r))e^{i\max (w_{ {\mathbb {R}} _{1}}(r),w_{ {\mathbb {R}} _{2}}(r))}. \end{aligned}$$

Now

30 $$\begin{aligned}. \left( {\mathbb {R}} _{1}^{c}\cup {\mathbb {R}} _{2}^{c}\right) \cap {\mathbb {R}} _{1}^{c}&=\{\max (1-\ell _{ {\mathbb {R}} _{1}}(r),1-\ell _{ {\mathbb {R}} _{2}}(r))e^{i\max (w_{ {\mathbb {R}} _{1}}(r),w_{ {\mathbb {R}} _{2}}(r))} \cap (1-\ell _{ {\mathbb {R}} _{1}}(r)e^{iw_{ {\mathbb {R}} _{1}}(r)})\}\nonumber \\&=\min \{\max (1-\ell _{ {\mathbb {R}} _{1}}(r),1-\ell _{ {\mathbb {R}} _{2}}(r)),(1-\ell _{ {\mathbb {R}} _{1}}(r)\} e^{i\min \{\max (w_{ {\mathbb {R}} _{1}}(r),w_{ {\mathbb {R}} _{2}}(r)),w_{ {\mathbb {R}} _{1}}(r)\}}. \end{aligned}$$

Now, to prove the identity (29), we are taken some cases: $\square$

Case 1. If $\ell _{ {\mathbb {R}} _{1}}(r)\le \ell _{ {\mathbb {R}} _{2}}(r)$ and $w_{ {\mathbb {R}} _{1}}(r)\le w_{ {\mathbb {R}} _{2}}(r)$ then (30) implies that

$$\begin{aligned} \left( {\mathbb {R}} _{1}^{c}\cup {\mathbb {R}} _{2}^{c}\right) \cap {\mathbb {R}} _{1}^{c}&=\min \{\max (1-\ell _{ {\mathbb {R}} _{1}}(r),1-\ell _{ {\mathbb {R}} _{2}}(r)),(1-\ell _{ {\mathbb {R}} _{1}}(r)\} e^{i\min \{\max (w_{ {\mathbb {R}} _{1}}(r),w_{ {\mathbb {R}} _{2}}(r)),w_{ {\mathbb {R}} _{1}}(r)\}},\\&=\min \{1-\ell _{ {\mathbb {R}} _{1}}(r),1-\ell _{ {\mathbb {R}} _{1}}(r)\}e^{i\min \{w_{ {\mathbb {R}} _{2}}(r)),w_{ {\mathbb {R}} _{1}}(r)\}}\\&=(1-\ell _{ {\mathbb {R}} _{1}}(r))e^{iw_{ {\mathbb {R}} _{1}}(r)}\\&= {\mathbb {R}} _{1}^{c}. \end{aligned}$$

Case 2. If $\ell _{ {\mathbb {R}} _{2}}(r)\le \ell _{ {\mathbb {R}} _{1}}(r)$ and $w_{ {\mathbb {R}} _{2}}(r)\le w_{ {\mathbb {R}} _{1}}(r)$ then

$$\begin{aligned} \left( {\mathbb {R}} _{1}^{c}\cup {\mathbb {R}} _{2}^{c}\right) \cap {\mathbb {R}} _{1}^{c}&=\min \{\max (1-\ell _{ {\mathbb {R}} _{1}}(r),1-\ell _{ {\mathbb {R}} _{2}}(r)),(1-\ell _{ {\mathbb {R}} _{1}}(r)\} e^{i\min \{\max (w_{ {\mathbb {R}} _{1}}(r),w_{ {\mathbb {R}} _{2}}(r)),w_{ {\mathbb {R}} _{1}}(r)\}},\\&=\min \{1-\ell _{ {\mathbb {R}} _{2}}(r),1-\ell _{ {\mathbb {R}} _{1}}(r)\}e^{i\min \{w_{ {\mathbb {R}} _{1}}(r)),w_{ {\mathbb {R}} _{1}}(r)\}}\\&=(1-\ell _{ {\mathbb {R}} _{1}}(r))e^{iw_{ {\mathbb {R}} _{1}}(r)}\\&= {\mathbb {R}} _{1}^{c}. \end{aligned}$$

The Proof is similar for other cases.

### Theorem 11

For $$ {\mathbb {R}} _{1}\ $$and ${\mathbb {R}} _{2}$ the CFSs on U, then CF standard union, standard intersection and complement satisfies the below result:

31 $$\begin{aligned} ( {\mathbb {R}} _{2}\cup ( {\mathbb {R}} _{2}\cap {\mathbb {R}} _{1}))= {\mathbb {R}} _{2}. \end{aligned}$$

### Proof

Let $\Im _{ {\mathbb {R}} _{1}}(r)=\ell _{ {\mathbb {R}} _{1}}(r)e^{iw_{ {\mathbb {R}} _{1}}(r)}$ and $\Im _{ {\mathbb {R}} _{2}(r)}=\ell _{ {\mathbb {R}} _{2}}(r)e^{iw_{ {\mathbb {R}} _{2}}(r)}$ are membership functions of $$ {\mathbb {R}} _{1}\ $$and ${\mathbb {R}} _{2},$ respectively. Then,

32 $$\begin{aligned} ( {\mathbb {R}} _{2}\cup ( {\mathbb {R}} _{2}\cap {\mathbb {R}} _{1})&=\Im _{ {\mathbb {R}} _{2}}(r)\cup (\Im _{ {\mathbb {R}} _{2}}(r)\cap \Im _{ {\mathbb {R}} _{1}}(r))\nonumber \\&=\max (\ell _{ {\mathbb {R}} _{2}}(r),\min \left( \ell _{ {\mathbb {R}} _{2}}(r),\ell _{ {\mathbb {R}} _{1}}(r)\right) e^{i\max \left( w_{ {\mathbb {R}} _{2}}(r),\min \left( w_{ {\mathbb {R}} _{2}}(r),w_{ {\mathbb {R}} _{1}}(r)\right) \right) }. \end{aligned}$$

To prove the given identity, we take some cases.

Case 1. If $\ell _{ {\mathbb {R}} _{1}}(r)\le \ell _{ {\mathbb {R}} _{2}}(r)$ and $w_{ {\mathbb {R}} _{1}}(r)\le w_{ {\mathbb {R}} _{2}}(r)$ then (32) implies that

$$\begin{aligned} ( {\mathbb {R}} _{2}\cup ( {\mathbb {R}} _{2}\cap {\mathbb {R}} _{1})&= \max (\ell _{ {\mathbb {R}} _{2}}(r),\min \left( \ell _{ {\mathbb {R}} _{2}}(r),\ell _{ {\mathbb {R}} _{1}}(r)\right) e^{i\max \left( w_{ {\mathbb {R}} _{2}}(r),\min \left( w_{ {\mathbb {R}} _{2}}(r),w_{ {\mathbb {R}} _{1}}(r)\right) \right) }\\&=\max (\ell _{ {\mathbb {R}} _{2}}(r),\ell _{ {\mathbb {R}} _{1}}(r))e^{i\max \left( w_{ {\mathbb {R}} _{2}}(r),w_{ {\mathbb {R}} _{1}}(r)\right) },\\&=\ell _{ {\mathbb {R}} _{2}}(r)e^{iw_{ {\mathbb {R}} _{2}}(r)},\\&= {\mathbb {R}} _{2}. \end{aligned}$$

Case 2. If $\ell _{ {\mathbb {R}} _{2}}(r)\le \ell _{ {\mathbb {R}} _{1}}(r)$ and $w_{ {\mathbb {R}} _{2}}(r)\le w_{ {\mathbb {R}} _{1}}(r)$ then (31) implies that

$$\begin{aligned} ( {\mathbb {R}} _{2}\cup ( {\mathbb {R}} _{2}\cap {\mathbb {R}} _{1})&=\max (\ell _{ {\mathbb {R}} _{2}}(r),\min \left( \ell _{ {\mathbb {R}} _{2}}(r),\ell _{ {\mathbb {R}} _{1}}(r)\right) e^{i\max \left( w_{ {\mathbb {R}} _{2}}(r),\min \left( w_{ {\mathbb {R}} _{2}}(r),w_{ {\mathbb {R}} _{1}}(r)\right) \right) }\\&=\max (\ell _{ {\mathbb {R}} _{2}}(r),\ell _{ {\mathbb {R}} _{2}}(r))e^{i\max \left( w_{ {\mathbb {R}} _{2}}(r),w_{ {\mathbb {R}} _{2}}(r)\right) },\\&=\ell _{ {\mathbb {R}} _{2}}(r)e^{iw_{ {\mathbb {R}} _{2}}(r)},\\&= {\mathbb {R}} _{2}. \end{aligned}$$

The Proof is similar for other cases. $\square$

## New complex fuzzy distance measures

The hesitance degree is often used in decision-making and fuzzy logic to represent uncertainty or indecision associated with a particular choice or preference. When considering a DM in conjunction with hesitance values, it typically relates to situations where preferences or similarities are not crisp and clear-cut but exist on a spectrum. Hesitance values can be incorporated into DMs in decision-making processes where multiple criteria are considered, and the decision-maker is hesitant or uncertain about the importance or weight of each criterion. In clustering algorithms, DMs with hesitance, values can be helpful when dealing with data points that don’t strictly belong to a single cluster but have a certain degree of membership in multiple clusters. When comparing strings or patterns, hesitance values can be used to indicate the uncertainty in the match. This is especially relevant in applications such as record linkage or information retrieval, where there might be partial matches or ambiguity. When combining DMs with hesitance values, it’s crucial to define a suitable metric that appropriately captures the uncertainty or hesitation in the context of the specific problem or application. This often involves using fuzzy logic techniques or similar approaches to handle imprecision and uncertainty in the data. However, the CFDMs with hesitance degree plays a significant role in decision-making, pattern recognition, medical diagnosis, human-computer interaction, and preference modeling.

Here, we define some new DMs with hesitance degree in the environment of CFSs, called complex fuzzy Hesitance distance measures and complex fuzzy Euclidean Hesitance distance measures. CFHDM and CFEHDM. These are generalizations of complex fuzzy normalized Hamming distance measures and complex fuzzy Euclidean distance measures. They enhance these measures by including the notion of hesitancy, providing a more nuanced understanding of data relationships.

### Complex fuzzy hesitance distance measures

#### Definition 17

A Hesitance Di-Me of CFSs is a function $d_{H}: {\mathbb {R}} ( {\mathcal {L}} )\times {\mathbb {R}} ( {\mathcal {L}} )\rightarrow [0,1]$ with the properties: for any ${\mathbb {R}} _{1},$ ${\mathbb {R}} _{2},$ ${\mathbb {R}} _{3}\in {\mathbb {R}} ( {\mathcal {L}} )($collection of CFSs):

(i).: $$0\le d_{H}( {\mathbb {R}} _{1},{\mathbb {R}} _{2})\le 1,$$ $$d_{H}( {\mathbb {R}} _{1},{\mathbb {R}} _{2})=0$$ $$\Longleftrightarrow $$ $$ {\mathbb {R}} _{1}= {\mathbb {R}} _{2}.$$
(ii).: $$d_{H}( {\mathbb {R}} _{1},{\mathbb {R}} _{2})=d_{H}( {\mathbb {R}} _{2},{\mathbb {R}} _{1}).$$
(iii).: $$d_{H}( {\mathbb {R}} _{1},{\mathbb {R}} _{3})\le d_{H}( {\mathbb {R}} _{1},{\mathbb {R}} _{2})+d_{H}( {\mathbb {R}} _{2},{\mathbb {R}} _{3}).$$

The Hesitance DMs is defined as:

38 $$\begin{aligned} d_{H}\left( {\mathbb {R}} _{1},{\mathbb {R}} _{2}\right)&=\frac{ {\displaystyle \sum _{i=1}^{n}} \left[ \begin{array}{c} \mid \ell _{ {\mathbb {R}} _{1}}\left( r_{i}\right) -\ell _{ {\mathbb {R}} _{2}}\left( r_{i}\right) \mid +\frac{1}{2\pi }\mid w_{ {\mathbb {R}} _{1}}\left( r_{i}\right) -w_{ {\mathbb {R}} _{2}}\left( r_{i}\right) \mid \\ +\mid \hslash _{ {\mathbb {R}} _{1}}\left( r_{i}\right) -\hslash _{ {\mathbb {R}} _{2}}\left( r_{i}\right) \mid \end{array} \right] }{3n}. \end{aligned}$$

#### Example 1

Let

$$\begin{aligned} {\mathbb {R}} _{1}&=\frac{0.3e^{i0.8\pi }}{r_{1}}+\frac{0.8e^{i0.4\pi }}{r_{2}} +\frac{0.3e^{i0.2\pi }}{r_{3}}+\frac{0.4e^{i1.8\pi }}{r_{2}}+\frac{0.9e^{i1\pi }}{r_{3}},\\ {\mathbb {R}} _{2}&=\frac{0.6e^{i0.4\pi }}{r_{1}}+\frac{0.1e^{i1.1\pi }}{r_{2}} +\frac{0.9e^{i1.5\pi }}{r_{3}}+\frac{0.2e^{i2\pi }}{r_{2}}+\frac{0.7e^{i0.6\pi }}{r_{3}}, \end{aligned}$$

then,

$$\begin{aligned} d_{H}\left( {\mathbb {R}} _{1},{\mathbb {R}} _{2}\right)&=\frac{\left[ \begin{array}{c} \left( |0.3-0.6|+\frac{1}{2\pi }|0.8\pi -0.4\pi |+|0.1-0.4|\right) +\\ \left( |0.8-0.1|+\frac{1}{2\pi }|0.4\pi -1.1\pi |+|0.6-0.45|\right) +\\ \left( |0.3-0.9|+\frac{1}{2\pi }|0.2\pi -1.5\pi |+|0.2-0.25|\right) +\\ \left( |0.4-0.2|+\frac{1}{2\pi }|1.8\pi -2\pi |+|0.5-0.8|\right) +\\ \left( |0.9-0.7|+\frac{1}{2\pi }|\pi -0.6\pi |+|0.4-0.4|\right) + \end{array} \right] }{3(5)},\\&=\frac{\left[ \begin{array}{c} 0.3+0.2+0.3+0.7+0.35+0.15+0.6+\\ 0.65+0.05+0.2+0.1+0.3+0.2+0.2+0.05 \end{array} \right] }{15},\\&=0.29. \end{aligned}$$

#### Theorem 12

The function $d_{H}$ defined in (38) is a distance function.

#### Proof

The condition $d_{H}\left( {\mathbb {R}} _{1},{\mathbb {R}} _{2}\right) \ge 0$ obviously holds true. Next consider

$$\begin{aligned} d_{H}\left( {\mathbb {R}} _{1},{\mathbb {R}} _{2}\right)&=\frac{ {\displaystyle \sum _{i=1}^{n}} \left[ \begin{array}{c} \mid \ell _{ {\mathbb {R}} _{1}}\left( r_{i}\right) -\ell _{ {\mathbb {R}} _{2}}\left( r_{i}\right) \mid +\frac{1}{2\pi }\mid w_{ {\mathbb {R}} _{1}}\left( r_{i}\right) -w_{ {\mathbb {R}} _{2}}\left( r_{i}\right) \mid \\ +\mid \hslash _{ {\mathbb {R}} _{1}}\left( r_{i}\right) -\hslash _{ {\mathbb {R}} _{2}}\left( r_{i}\right) \mid \end{array} \right] }{3n}\\&=\frac{ {\displaystyle \sum _{i=1}^{n}} [1+1+1]}{3n} \end{aligned}$$

$$\begin{aligned}&=\left[ \frac{3}{3n}\right] \\&=1. \end{aligned}$$

Therefore $$0\le d_{H}\left( {\mathbb {R}} _{1},{\mathbb {R}} _{2}\right) \le 1.$$ (ii):

$$\begin{aligned} d_{H}\left( {\mathbb {R}} _{1},{\mathbb {R}} _{2}\right)&=\frac{ {\displaystyle \sum _{i=1}^{n}} \left[ \begin{array}{c} \mid \ell _{ {\mathbb {R}} _{1}}\left( r_{i}\right) -\ell _{ {\mathbb {R}} _{2}}\left( r_{i}\right) \mid +\frac{1}{2\pi }\mid w_{ {\mathbb {R}} _{1}}\left( r_{i}\right) -w_{ {\mathbb {R}} _{2}}\left( r_{i}\right) \mid \\ +\left[ \mid \hslash _{ {\mathbb {R}} _{1}}\left( r_{i}\right) -\hslash _{ {\mathbb {R}} _{2}}\left( r_{i}\right) \mid \right] \end{array} \right] }{3n},\\&=\frac{ {\displaystyle \sum _{i=1}^{n}} \left[ \begin{array}{c} \mid \ell _{ {\mathbb {R}} _{2}}\left( r_{i}\right) -\ell _{ {\mathbb {R}} _{1}}\left( r_{i}\right) \mid +\frac{1}{2\pi }\mid w_{ {\mathbb {R}} _{2}}\left( r_{i}\right) -w_{ {\mathbb {R}} _{1}}\left( r_{i}\right) \mid \\ +\mid \hslash _{ {\mathbb {R}} _{2}}\left( r_{i}\right) -\hslash _{ {\mathbb {R}} _{1}}\left( r_{i}\right) \mid \end{array} \right] }{3n},\\&=d_{H}\left( {\mathbb {R}} _{2},{\mathbb {R}} _{1}\right) . \end{aligned}$$

(iii).

$$\begin{aligned} d_{H}\left( {\mathbb {R}} _{1},{\mathbb {R}} _{2}\right)&=\frac{ {\displaystyle \sum _{i=1}^{n}} \left[ \begin{array}{c} \mid \ell _{ {\mathbb {R}} _{1}}\left( r_{i}\right) -\ell _{ {\mathbb {R}} _{2}}\left( r_{i}\right) \mid +\frac{1}{2\pi }\mid w_{ {\mathbb {R}} _{1}}\left( r_{i}\right) -w_{ {\mathbb {R}} _{2}}\left( r_{i}\right) \mid \\ +\mid \hslash _{ {\mathbb {R}} _{1}}\left( r_{i}\right) -\hslash _{ {\mathbb {R}} _{2}}\left( r_{i}\right) \mid \end{array} \right] }{3n},\\&=\frac{ {\displaystyle \sum _{i=1}^{n}} \left[ \begin{array}{c} \mid \ell _{ {\mathbb {R}} _{1}}\left( r_{i}\right) -\ell _{ {\mathbb {R}} _{3}}\left( r_{i}\right) +\ell _{ {\mathbb {R}} _{3}}\left( r_{i}\right) -\ell _{ {\mathbb {R}} _{2}}\left( r_{i}\right) \mid +\\ \frac{1}{2\pi }\mid w_{ {\mathbb {R}} _{1}}\left( r_{i}\right) -w_{ {\mathbb {R}} _{3}}\left( r_{i}\right) +w_{ {\mathbb {R}} _{3}}\left( r_{i}\right) -w_{ {\mathbb {R}} _{2}}\left( r_{i}\right) \mid +\\ \mid \hslash _{ {\mathbb {R}} _{1}}\left( r_{i}\right) -\hslash _{ {\mathbb {R}} _{3}}\left( r_{i}\right) +\hslash _{ {\mathbb {R}} _{3}}\left( r_{i}\right) -\hslash _{ {\mathbb {R}} _{2}}\left( r_{i}\right) \mid \end{array} \right] }{3n},\\&\le \frac{ {\displaystyle \sum _{i=1}^{n}} \left[ \begin{array}{c} \mid \ell _{ {\mathbb {R}} _{1}}\left( r_{i}\right) -\ell _{ {\mathbb {R}} _{3}}\left( r_{i}\right) \mid +\mid \ell _{ {\mathbb {R}} _{3}}\left( r_{i}\right) -\ell _{ {\mathbb {R}} _{2}}\left( r_{i}\right) \mid +\\ \frac{1}{2\pi }\mid w_{ {\mathbb {R}} _{1}}\left( r_{i}\right) -w_{ {\mathbb {R}} _{3}}\left( r_{i}\right) \mid +\mid w_{ {\mathbb {R}} _{3}}\left( r_{i}\right) -w_{ {\mathbb {R}} _{2}}\left( r_{i}\right) \mid +\\ \mid \hslash _{ {\mathbb {R}} _{1}}\left( r_{i}\right) -\hslash _{ {\mathbb {R}} _{3}}\left( r_{i}\right) \mid +\mid \hslash _{ {\mathbb {R}} _{3}}\left( r_{i}\right) -\hslash _{ {\mathbb {R}} _{2}}\left( r_{i}\right) \mid \end{array} \right] }{3n}\\&=\frac{ {\displaystyle \sum _{i=1}^{n}} \left[ \begin{array}{c} \mid \ell _{ {\mathbb {R}} _{1}}\left( r_{i}\right) -\ell _{ {\mathbb {R}} _{3}}\left( r_{i}\right) \mid +\frac{1}{2\pi }\mid w_{ {\mathbb {R}} _{1}}\left( r_{i}\right) -w_{ {\mathbb {R}} _{3}}\left( r_{i}\right) \mid \\ +\mid \hslash _{ {\mathbb {R}} _{1}}\left( r_{i}\right) -\hslash _{ {\mathbb {R}} _{3}}\left( r_{i}\right) \mid \end{array} \right] }{3n}+\\&\quad \frac{ {\displaystyle \sum _{i=1}^{n}} \left[ \begin{array}{c} \mid \ell _{ {\mathbb {R}} _{2}}\left( r_{i}\right) -\ell _{ {\mathbb {R}} _{3}}\left( r_{i}\right) \mid +\frac{1}{2\pi }\mid w_{ {\mathbb {R}} _{2}}\left( r_{i}\right) -w_{ {\mathbb {R}} _{3}}\left( r_{i}\right) \mid \\ +\mid \hslash _{ {\mathbb {R}} _{2}}\left( r_{i}\right) -\hslash _{ {\mathbb {R}} _{3}}\left( r_{i}\right) \mid \end{array} \right] }{3n}\\&=d_{H}\left( {\mathbb {R}} _{1},{\mathbb {R}} _{2}\right) +d_{H}\left( {\mathbb {R}} _{1},{\mathbb {R}} _{2}\right) . \end{aligned}$$

$$\square $$

#### Definition 18

A Euclidean Hesitance Di-Me of CFSs is a function $d_{EH}: {\mathbb {R}} ( {\mathcal {L}} )\times {\mathbb {R}} ( {\mathcal {L}} )\rightarrow [0,1]$ with the properties: for any ${\mathbb {R}} _{1},$ ${\mathbb {R}} _{2},$ ${\mathbb {R}} _{3}\in {\mathbb {R}} ( {\mathcal {L}} )($collection of CFSs):

(i).: $$0\le d_{EH}( {\mathbb {R}} _{1},{\mathbb {R}} _{2})\le 1,$$
(ii).: $$d_{EH}( {\mathbb {R}} _{1},{\mathbb {R}} _{2})=0$$ $$\Longleftrightarrow $$ $$ {\mathbb {R}} _{1}= {\mathbb {R}} _{2}.$$
(iii).: $$d_{EH}( {\mathbb {R}} _{1},{\mathbb {R}} _{2})=d_{EH}( {\mathbb {R}} _{2},{\mathbb {R}} _{1}).$$
(iv).: $$d_{EH}( {\mathbb {R}} _{1},{\mathbb {R}} _{3})\le d_{EH}( {\mathbb {R}} _{1},{\mathbb {R}} _{2})+d_{EH}( {\mathbb {R}} _{2},{\mathbb {R}} _{3}).$$

The Hesitance Di-Mes is defined as:

39 $$\begin{aligned} d_{EH}\left( {\mathbb {R}} _{1},{\mathbb {R}} _{2}\right) =\sqrt{\frac{ {\displaystyle \sum _{i=1}^{n}} \left[ \begin{array}{c} (\ell _{ {\mathbb {R}} _{1}}\left( r_{i}\right) -\ell _{ {\mathbb {R}} _{2}}\left( r_{i}\right) )^{2}+\frac{1}{2\pi ^{2}}(w_{ {\mathbb {R}} _{1}}\left( r_{i}\right) -w_{ {\mathbb {R}} _{2}}\left( r_{i}\right) )^{2}+\\ (\hslash _{ {\mathbb {R}} _{1}}\left( r_{i}\right) -\hslash _{ {\mathbb {R}} _{2}}\left( r_{i}\right) )^{2} \end{array} \right] }{3n}}. \end{aligned}$$

#### Example 2

Let

$$\begin{aligned} {\mathbb {R}} _{1}&=\frac{0.3e^{i0.8\pi }}{r_{1}}+\frac{0.8e^{i0.4\pi }}{r_{2}} +\frac{0.3e^{i0.2\pi }}{r_{3}},\\{\mathbb {R}} _{2}&=\frac{0.6e^{i0.4\pi }}{r_{1}}+\frac{0.1e^{i1.1\pi }}{r_{2}} +\frac{0.9e^{i1.5\pi }}{r_{3}}, \end{aligned}$$

then,

$$\begin{aligned} d_{EH}\left( {\mathbb {R}} _{1},{\mathbb {R}} _{2}\right)&=\left[ \frac{ \begin{array}{c} \left( (0.3-0.6)^{2}+\frac{1}{2\pi ^{2}}(0.8\pi -0.4\pi )^{2}+(0.1-0.4)^{2} \right) +\\ \left( (0.8-0.1)^{2}+\frac{1}{2\pi ^{2}}(0.4\pi -1.1\pi )^{2}+(0.6-0.45)^{2} \right) +\\ \left( (0.3-0.9)^{2}+\frac{1}{2\pi ^{2}}(0.2\pi -1.5\pi )^{2}+(0.2-0.25)^{2} \right) + \end{array} }{3(3)}\right] ^{\frac{1}{2}},\\&=\left[ \frac{ \begin{array}{c} 0.09+0.04+0.09+0.49+0.125+0.03+\\ 0.36+0.42+0.01 \end{array} }{9}\right] ^{\frac{1}{2}},\\&=0.4. \end{aligned}$$

#### Theorem 13

The function $d_{EH}$ defined in (39) is a distance function.

#### Proof

The condition $d_{EH}\left( {\mathbb {R}} _{1},{\mathbb {R}} _{2}\right) \ge 0$ obviously holds true. Next, consider

$$\begin{aligned} d_{EH}\left( {\mathbb {R}} _{1},{\mathbb {R}} _{2}\right)&=\sqrt{\frac{ {\displaystyle \sum _{i=1}^{n}} \left[ \begin{array}{c} (\ell _{ {\mathbb {R}} _{1}}\left( r_{i}\right) -\ell _{ {\mathbb {R}} _{2}}\left( r_{i}\right) )^{2}+\frac{1}{2\pi ^{2}}(w_{ {\mathbb {R}} _{1}}\left( r_{i}\right) -w_{ {\mathbb {R}} _{2}}\left( r_{i}\right) )^{2}+\\ (\hslash _{ {\mathbb {R}} _{1}}\left( r_{i}\right) -\hslash _{ {\mathbb {R}} _{2}}\left( r_{i}\right) )^{2} \end{array} \right] }{3n}},\\&=\sqrt{ {\displaystyle \sum _{i=1}^{n}} \frac{(1+1+1)}{3n}}=\sqrt{\frac{1}{n}}. \end{aligned}$$

If $n=1$ then $d_{EH}\left( {\mathbb {R}} _{1},{\mathbb {R}} _{2}\right) =1$ otherwise $d_{EH}\left( {\mathbb {R}} _{1},{\mathbb {R}} _{2}\right) <1.$Therefore $0\le d_{EH}\left( {\mathbb {R}} _{1},{\mathbb {R}} _{2}\right) \le 1.$

(ii).

$$\begin{aligned} d_{EH}\left( {\mathbb {R}} _{1},{\mathbb {R}} _{2}\right)&=\sqrt{\frac{ {\displaystyle \sum _{i=1}^{n}} \left[ \begin{array}{c} (\ell _{ {\mathbb {R}} _{1}}\left( r_{i}\right) -\ell _{ {\mathbb {R}} _{2}}\left( r_{i}\right) )^{2}+\frac{1}{2\pi ^{2}}(w_{ {\mathbb {R}} _{1}}\left( r_{i}\right) -w_{ {\mathbb {R}} _{2}}\left( r_{i}\right) )^{2}+\\ (\hslash _{ {\mathbb {R}} _{1}}\left( r_{i}\right) -\hslash _{ {\mathbb {R}} _{2}}\left( r_{i}\right) )^{2} \end{array} \right] }{3n}},\\&=\sqrt{\frac{ {\displaystyle \sum _{i=1}^{n}} \left[ \begin{array}{c} (\ell _{ {\mathbb {R}} _{2}}\left( r_{i}\right) -\ell _{ {\mathbb {R}} _{1}}\left( r_{i}\right) )^{2}+\frac{1}{2\pi ^{2}}(w_{ {\mathbb {R}} _{2}}\left( r_{i}\right) -w_{ {\mathbb {R}} _{1}}\left( r_{i}\right) )^{2}+\\ (\hslash _{ {\mathbb {R}} _{2}}\left( r_{ii}\right) -\hslash _{ {\mathbb {R}} _{1}}\left( r_{i}\right) )^{2} \end{array} \right] }{3n}},\\&=d_{EH}\left( {\mathbb {R}} _{2},{\mathbb {R}} _{1}\right) . \end{aligned}$$

(iii).

$$\begin{aligned} d_{EH}\left( {\mathbb {R}} _{1},{\mathbb {R}} _{2}\right)&=\sqrt{\frac{ {\displaystyle \sum _{i=1}^{n}} \left[ \begin{array}{c} (\ell _{ {\mathbb {R}} _{1}}\left( r_{i}\right) -\ell _{ {\mathbb {R}} _{2}}\left( r_{i}\right) )^{2}+\frac{1}{2\pi ^{2}}(w_{ {\mathbb {R}} _{1}}\left( r_{i}\right) -w_{ {\mathbb {R}} _{2}}\left( r_{i}\right) )^{2}+\\ (\hslash _{ {\mathbb {R}} _{1}}\left( r_{i}\right) -\hslash _{ {\mathbb {R}} _{2}}\left( r_{i}\right) )^{2} \end{array} \right] }{3n}},\\&=\sqrt{\frac{ {\displaystyle \sum _{i=1}^{n}} \left[ \begin{array}{c} (\ell _{ {\mathbb {R}} _{1}}\left( r_{i}\right) -\ell _{ {\mathbb {R}} _{3}}\left( r_{i}\right) +\ell _{ {\mathbb {R}} _{3}}\left( r_{i}\right) -\ell _{ {\mathbb {R}} _{2}}\left( r_{i}\right) )^{2}+\\ \frac{1}{2\pi ^{2}}\left( w_{ {\mathbb {R}} _{1}}\left( r_{i}\right) -w_{ {\mathbb {R}} _{3}}\left( r_{i}\right) +w_{ {\mathbb {R}} _{3}}\left( r_{i}\right) -w_{ {\mathbb {R}} _{2}}\left( r_{i}\right) \right) ^{2}+\\ (\hslash _{ {\mathbb {R}} _{1}}\left( r_{i}\right) -\hslash _{ {\mathbb {R}} _{3}}\left( r_{i}\right) +\hslash _{ {\mathbb {R}} _{3}}\left( r_{i}\right) -\hslash _{ {\mathbb {R}} _{2}}\left( r_{i}\right) )^{2} \end{array} \right] }{3n}}\\&\quad \sqrt{\frac{ {\displaystyle \sum _{i=1}^{n}} \left[ \begin{array}{c} (\ell _{ {\mathbb {R}} _{1}}\left( r_{i}\right) -\ell _{ {\mathbb {R}} _{3}}\left( r_{i}\right) )^{2}+\frac{1}{2\pi ^{2}}(w_{ {\mathbb {R}} _{1}}\left( r_{i}\right) -w_{ {\mathbb {R}} _{3}}\left( r_{i}\right) )^{2}+\\ (\hslash _{ {\mathbb {R}} _{1}}\left( r_{i}\right) -\hslash _{ {\mathbb {R}} _{3}}\left( r_{i}\right) )^{2} \end{array} \right] }{3n}}+\\&\quad \sqrt{\frac{ {\displaystyle \sum _{i=1}^{n}} \left[ \begin{array}{c} (\ell _{ {\mathbb {R}} _{3}}\left( r_{i}\right) -\ell _{ {\mathbb {R}} _{2}}\left( r_{i}\right) )^{2}+\frac{1}{2\pi ^{2}}(w_{ {\mathbb {R}} _{3}}\left( r_{i}\right) -w_{ {\mathbb {R}} _{2}}\left( r_{i}\right) )^{2}+\\ (\hslash _{ {\mathbb {R}} _{3}}\left( r_{i}\right) -\hslash _{ {\mathbb {R}} _{2}}\left( r_{i}\right) )^{2} \end{array} \right] }{3n}}\\&\quad \text {(by Minkowski's Inequality)}\\ d_{EH}\left( {\mathbb {R}} _{1},{\mathbb {R}} _{2}\right)&\le d_{EH}\left( {\mathbb {R}} _{1},{\mathbb {R}} _{3}\right) +d_{EH}\left( {\mathbb {R}} _{3},{\mathbb {R}} _{2}\right) . \end{aligned}$$

$$\square $$

#### Definition 19

Let $d_{H}$ be the Hesitance measure of CFSs Then,the complement Hesitance distance measure $d_{H}(\left( {\mathbb {R}} _{1})^{c},( {\mathbb {R}} _{2}\right) ^{c})$ of two CFS is defined as

$$\begin{aligned} d_{H}(\left( {\mathbb {R}} _{1})^{c},( {\mathbb {R}} _{2}\right) ^{c})=\frac{ {\displaystyle \sum _{i=1}^{n}} \left[ \begin{array}{c} \mid (1-\ell _{ {\mathbb {R}} _{1}}\left( r_{i}\right) )-(1-\ell _{ {\mathbb {R}} _{2}}\left( r_{i}\right) )\mid +\\ \frac{1}{2\pi }\mid w_{ {\mathbb {R}} _{1}}\left( r_{i}\right) -w_{ {\mathbb {R}} _{2}}\left( r_{i}\right) \mid \\ +\mid (1-\hslash _{ {\mathbb {R}} _{1}}\left( r_{i}\right) )-(1-\hslash _{ {\mathbb {R}} _{2}}\left( r_{i}\right) )\mid \end{array} \right] }{3n}. \end{aligned}$$

#### Definition 20

Let $d_{H}$ be the Hesitance measure of CFSs Then,the max Hesitance distance measure of two CFSs ${\mathbb {R}} _{1}$and ${\mathbb {R}} _{2}$ is denoted by $d_{H}( {\mathbb {R}} _{1}\cup {\mathbb {R}} _{2},{\mathbb {R}} _{2})$ or $d_{H}( {\mathbb {R}} _{1},{\mathbb {R}} _{2}\cup {\mathbb {R}} _{2})$ and satisfied the function

$$\begin{aligned} d_{H}( {\mathbb {R}} _{1}\cup {\mathbb {R}} _{2},{\mathbb {R}} _{2})=\frac{ {\displaystyle \sum _{i=1}^{n}} \left[ \begin{array}{c} \mid \left( \ell _{ {\mathbb {R}} _{1}}\left( r_{i}\right) \vee \ell _{ {\mathbb {R}} _{2}}\left( r_{i}\right) \right) -\ell _{ {\mathbb {R}} _{2}}\left( r_{i}\right) \mid +\\ \frac{1}{2\pi }\mid \left( w_{ {\mathbb {R}} _{1}}\left( r_{i}\right) \vee w_{ {\mathbb {R}} _{2}}\left( r_{i}\right) \right) -w_{ {\mathbb {R}} _{2}}\left( r_{i}\right) \mid \\ +\mid \left( \hslash _{ {\mathbb {R}} _{1}}\left( r_{i}\right) \vee \hslash _{ {\mathbb {R}} _{2}}\left( r_{i}\right) \right) -\hslash _{ {\mathbb {R}} _{2}}\left( r_{i}\right) \mid \end{array} \right] }{3n}, \end{aligned}$$

and

$$\begin{aligned} d_{H}( {\mathbb {R}} _{1},{\mathbb {R}} _{2}\cup {\mathbb {R}} _{2})=\frac{ {\displaystyle \sum _{i=1}^{n}} \left[ \begin{array}{c} \mid \ell _{ {\mathbb {R}} _{1}}\left( r_{i}\right) -\left( \ell _{ {\mathbb {R}} _{2}}\left( r_{i}\right) \vee \ell _{ {\mathbb {R}} _{2}}\left( r_{i}\right) \right) +\\ \frac{1}{2\pi }\mid w_{ {\mathbb {R}} _{1}}\left( r_{i}\right) -\left( w_{ {\mathbb {R}} _{2}}\left( r_{i}\right) \vee w_{ {\mathbb {R}} _{2}}\left( r_{i}\right) \right) \mid \\ +\mid \hslash _{ {\mathbb {R}} _{1}}\left( r_{i}\right) -\left( \hslash _{ {\mathbb {R}} _{2}}\left( r_{i}\right) \vee \hslash _{ {\mathbb {R}} _{2}}\left( r_{i}\right) \right) \mid \end{array} \right] }{3n}, \end{aligned}$$

where $\vee$ denotes the max operator of CFSs.

#### Definition 21

Let $d_{H}$ be the Hesitance measure of CFSs Then,the min Hesitance distance measure of two CFSs ${\mathbb {R}} _{1}$and ${\mathbb {R}} _{2}$ is denoted by $d_{H}( {\mathbb {R}} _{1}\cap {\mathbb {R}} _{2},{\mathbb {R}} _{2})$ or $d_{H}( {\mathbb {R}} _{1},{\mathbb {R}} _{2}\cap {\mathbb {R}} _{2})$ and satisfied the function

$$\begin{aligned} d_{H}( {\mathbb {R}} _{1}\cap {\mathbb {R}} _{2},{\mathbb {R}} _{2})=\frac{ {\displaystyle \sum _{i=1}^{n}} \left[ \begin{array}{c} \left( \ell _{ {\mathbb {R}} _{1}}\left( r_{i}\right) \wedge \ell _{ {\mathbb {R}} _{2}}\left( r_{i}\right) \right) -\ell _{ {\mathbb {R}} _{2}}\left( r_{i}\right) \mid +\\ \frac{1}{2\pi }\mid \left( w_{ {\mathbb {R}} _{1}}\left( r_{i}\right) \wedge w_{ {\mathbb {R}} _{2}}\left( r_{i}\right) \right) -w_{ {\mathbb {R}} _{2}}\left( r_{i}\right) \mid +\\ \mid \left( \hslash _{ {\mathbb {R}} _{1}}\left( r_{i}\right) \wedge \hslash _{ {\mathbb {R}} _{2}}\left( r_{i}\right) \right) -\hslash _{ {\mathbb {R}} _{2}}\left( r_{i}\right) \mid \end{array} \right] }{3n}, \end{aligned}$$

and

$$\begin{aligned} d_{H}( {\mathbb {R}} _{1},{\mathbb {R}} _{2}\cap {\mathbb {R}} _{2})=\frac{ {\displaystyle \sum _{i=1}^{n}} \left[ \begin{array}{c} \mid \ell _{ {\mathbb {R}} _{1}}\left( r_{i}\right) -\left( \ell _{ {\mathbb {R}} _{2}}\left( r_{i}\right) \wedge \ell _{ {\mathbb {R}} _{2}}\left( r_{i}\right) \right) \mid +\\ \frac{1}{2\pi }\mid w_{ {\mathbb {R}} _{1}}\left( r_{i}\right) -\left( w_{ {\mathbb {R}} _{2}}\left( r_{i}\right) \wedge w_{ {\mathbb {R}} _{2}}\left( r_{i}\right) \right) \mid +\\ \mid \hslash _{ {\mathbb {R}} _{1}}\left( r_{i}\right) -\left( \hslash _{ {\mathbb {R}} _{2}}\left( r_{i}\right) \wedge \hslash _{ {\mathbb {R}} _{2}}\left( r_{i}\right) \right) \mid \end{array} \right] }{3n}. \end{aligned}$$

Here, $\wedge$ denotes the min operator of CFSs.

#### Definition 22

Let $d_{H}^{\prime }$ be a Hesitance DM of CFSs. Then,the max-min-Hesitance DM of two CFSs ${\mathbb {R}} _{1}$ and ${\mathbb {R}} _{2},$denoted by $d_{H}^{\prime }( {\mathbb {R}} _{1}\cup {\mathbb {R}} _{2},{\mathbb {R}} _{1}\cap {\mathbb {R}} _{2})$ or $d_{H}^{\prime }( {\mathbb {R}} _{1}\cap {\mathbb {R}} _{2},{\mathbb {R}} _{1}\cup {\mathbb {R}} _{2})$ is specified by the function

$$\begin{aligned} d_{H}( {\mathbb {R}} _{1}\cup {\mathbb {R}} _{2},{\mathbb {R}} _{1}\cap {\mathbb {R}} _{2})=\frac{ {\displaystyle \sum _{i=1}^{n}} \left[ \begin{array}{c} \mid \left( \ell _{ {\mathbb {R}} _{1}}\left( r_{i}\right) \vee \ell _{ {\mathbb {R}} _{2}}\left( r_{i}\right) \right) -\left( \ell _{ {\mathbb {R}} _{1}}\left( r_{i}\right) \wedge \ell _{ {\mathbb {R}} _{2}}\left( r_{i}\right) \right) \mid +\\ \frac{1}{2\pi }\mid \left( w_{ {\mathbb {R}} _{1}}\left( r_{i}\right) \vee w_{ {\mathbb {R}} _{2}}\left( r_{i}\right) \right) -\left( w_{ {\mathbb {R}} _{1}}\left( r_{i}\right) \wedge w_{ {\mathbb {R}} _{2}}\left( r_{i}\right) \right) \mid +\\ \mid \left( \hslash _{ {\mathbb {R}} _{1}}\left( r_{i}\right) \vee \hslash _{ {\mathbb {R}} _{2}}\left( r_{i}\right) \right) -\left( \hslash _{ {\mathbb {R}} _{1}}\left( r_{i}\right) \wedge \hslash _{ {\mathbb {R}} _{2}}\left( r_{i}\right) \right) \mid \end{array} \right] }{3n}, \end{aligned}$$

and

$$\begin{aligned} d_{H}( {\mathbb {R}} _{1}\cap {\mathbb {R}} _{2},{\mathbb {R}} _{1}\cup {\mathbb {R}} _{2})=\frac{ {\displaystyle \sum _{i=1}^{n}} \left[ \begin{array}{c} \mid \left( \ell _{ {\mathbb {R}} _{1}}\left( r_{i}\right) \wedge \ell _{ {\mathbb {R}} _{2}}\left( r_{i}\right) \right) -\left( \ell _{ {\mathbb {R}} _{1}}\left( r_{i}\right) \vee \ell _{ {\mathbb {R}} _{2}}\left( r_{i}\right) \right) \mid +\\ \frac{1}{2\pi }\mid \left( w_{ {\mathbb {R}} _{1}}\left( r_{i}\right) \wedge w_{ {\mathbb {R}} _{2}}\left( r_{i}\right) \right) -\left( w_{ {\mathbb {R}} _{1}}\left( r_{i}\right) \vee w_{ {\mathbb {R}} _{2}}\left( r_{i}\right) \right) \mid +\\ \mid \left( \hslash _{ {\mathbb {R}} _{1}}\left( r_{i}\right) \wedge \hslash _{ {\mathbb {R}} _{2}}\left( r_{i}\right) \right) -\left( \hslash _{ {\mathbb {R}} _{1}}\left( r_{i}\right) \vee \hslash _{ {\mathbb {R}} _{2}}\left( r_{i}\right) \right) \mid \end{array} \right] }{3n}, \end{aligned}$$

where $\vee$ and $\wedge$ denote max and min, complex fuzzy operators.

#### Definition 23

Let $d_{EH}$ be the Euclidean Hesitance measure of CFSs Then,the complement Euclidean Hesitance distance measure $d_{EH}(\left( {\mathbb {R}} _{1})^{c},( {\mathbb {R}} _{2}\right) ^{c})$ of two CFS is defined as

$$\begin{aligned} d_{EH}(\left( {\mathbb {R}} _{1})^{c},( {\mathbb {R}} _{2}\right) ^{c})=\sqrt{\frac{ {\displaystyle \sum _{i=1}^{n}} \left[ \begin{array}{c} ((1-\ell _{ {\mathbb {R}} _{1}}\left( r_{i}\right) )^{2}-(1-\ell _{ {\mathbb {R}} _{2}}\left( r_{i}\right) )^{2}+\\ \frac{1}{2\pi ^{2}}(w_{ {\mathbb {R}} _{1}}\left( r_{i}\right) -w_{ {\mathbb {R}} _{2}}\left( r_{i}\right) )^{2}+\\ ((1-\hslash _{ {\mathbb {R}} _{1}}\left( r_{i}\right) )^{2}-(1-\hslash _{ {\mathbb {R}} _{2}}\left( r_{i}\right) )^{2} \end{array} \right] }{3n}}. \end{aligned}$$

#### Definition 24

Let $d_{EH}$ be EuclideanHesitance measure of CFSs Then,the max Euclidean Hesitance distance measure of two CFSs ${\mathbb {R}} _{1}$and ${\mathbb {R}} _{2}$ is denoted by $d_{EH}( {\mathbb {R}} _{1}\cup {\mathbb {R}} _{2},{\mathbb {R}} _{2})$ or $d_{EH}( {\mathbb {R}} _{1},{\mathbb {R}} _{2}\cup {\mathbb {R}} _{2})$ and satisfied the function

$$\begin{aligned} d_{EH}\left( {\mathbb {R}} _{1}\cup {\mathbb {R}} _{2},{\mathbb {R}} _{2}\right) =\sqrt{\frac{ {\displaystyle \sum _{i=1}^{n}} \left[ \begin{array}{c} (\left( \ell _{ {\mathbb {R}} _{1}}\left( r_{i}\right) \vee \ell _{ {\mathbb {R}} _{2}}\left( r_{i}\right) \right) -\ell _{ {\mathbb {R}} _{2}}\left( r_{i}\right) )^{2}+\\ \frac{1}{2\pi ^{2}}(\left( w_{ {\mathbb {R}} _{1}}\left( r_{i}\right) \vee w_{ {\mathbb {R}} _{2}}\left( r_{i}\right) \right) -w_{ {\mathbb {R}} _{2}}\left( r_{i}\right) )^{2}+\\ ((\hslash _{ {\mathbb {R}} _{1}}\left( r_{i}\right) \vee \hslash _{ {\mathbb {R}} _{2}}\left( r_{i}\right) )-\hslash _{ {\mathbb {R}} _{2}}\left( r_{i}\right) )^{2} \end{array} \right] }{3n}} \end{aligned}$$

and

$$\begin{aligned} d_{EH}( {\mathbb {R}} _{1},{\mathbb {R}} _{2}\cup {\mathbb {R}} _{2})=\sqrt{\frac{ {\displaystyle \sum _{i=1}^{n}} \left[ \begin{array}{c} (\ell _{ {\mathbb {R}} _{1}}\left( r_{i}\right) -\left( \ell _{ {\mathbb {R}} _{2}}\left( r_{i}\right) \vee \ell _{ {\mathbb {R}} _{2}}\left( r_{i}\right) \right) )^{2}+\\ \frac{1}{2\pi ^{2}}(w_{ {\mathbb {R}} _{1}}\left( r_{i}\right) -\left( w_{ {\mathbb {R}} _{2}}\left( r_{i}\right) \vee w_{ {\mathbb {R}} _{2}}\left( r_{i}\right) \right) )^{2}+\\ (\hslash _{ {\mathbb {R}} _{1}}\left( r_{i}\right) -\left( \hslash _{ {\mathbb {R}} _{2}}\left( r_{i}\right) \vee \hslash _{ {\mathbb {R}} _{2}}\left( r_{i}\right) \right) )^{2} \end{array} \right] }{3n}}, \end{aligned}$$

where $\vee$ represents the max operator of CFSs.

#### Definition 25

Let $d_{EH}$ be the Euclidean Hesitance measure of CFSs Then,the min Euclidean Hesitance DM of two CFSs ${\mathbb {R}} _{1}$and ${\mathbb {R}} _{2}$ is denoted by $d_{EH}( {\mathbb {R}} _{1}\cap {\mathbb {R}} _{2},{\mathbb {R}} _{2})$ or $d_{EH}( {\mathbb {R}} _{1},{\mathbb {R}} _{2}\cap {\mathbb {R}} _{2})$ and satisfies the function.

$$\begin{aligned} d_{EH}\left( {\mathbb {R}} _{1}\cap {\mathbb {R}} _{2},{\mathbb {R}} _{2}\right) =\sqrt{\frac{ {\displaystyle \sum _{i=1}^{n}} \left[ \begin{array}{c} (\left( \ell _{ {\mathbb {R}} _{1}}\left( r_{i}\right) \wedge \ell _{ {\mathbb {R}} _{2}}\left( r_{i}\right) \right) -\ell _{ {\mathbb {R}} _{2}}\left( r_{i}\right) )^{2}+\\ \frac{1}{2\pi ^{2}}(\left( w_{ {\mathbb {R}} _{1}}\left( r_{i}\right) \wedge w_{ {\mathbb {R}} _{2}}\left( r_{i}\right) \right) -w_{ {\mathbb {R}} _{2}}\left( r_{i}\right) )^{2}+\\ ((\hslash _{ {\mathbb {R}} _{1}}\left( r_{i}\right) \wedge \hslash _{ {\mathbb {R}} _{2}}\left( r_{i}\right) )-\hslash _{ {\mathbb {R}} _{2}}\left( r_{i}\right) )^{2} \end{array} \right] }{3n}} \end{aligned}$$

and

$$\begin{aligned} d_{EH}( {\mathbb {R}} _{1},{\mathbb {R}} _{2}\cap {\mathbb {R}} _{2})=\sqrt{\frac{ {\displaystyle \sum _{i=1}^{n}} \left[ \begin{array}{c} (\ell _{ {\mathbb {R}} _{1}}\left( r_{i}\right) -\left( \ell _{ {\mathbb {R}} _{2}}\left( r_{i}\right) \wedge \ell _{ {\mathbb {R}} _{2}}\left( r_{i}\right) \right) )^{2}+\\ \frac{1}{2\pi ^{2}}(w_{ {\mathbb {R}} _{1}}\left( r_{i}\right) -\left( w_{ {\mathbb {R}} _{2}}\left( r_{i}\right) \wedge w_{ {\mathbb {R}} _{2}}\left( r_{i}\right) \right) )^{2}+\\ (\hslash _{ {\mathbb {R}} _{1}}\left( r_{i}\right) -\left( \hslash _{ {\mathbb {R}} _{2}}\left( r_{i}\right) \wedge \hslash _{ {\mathbb {R}} _{2}}\left( r_{i}\right) \right) )^{2} \end{array} \right] }{3n}}. \end{aligned}$$

Here, $\wedge$ denotes the min-operator.

#### Definition 26

Let $d_{EH}$ be Euclidean Hacitance DM of CFSs. Then, the max-min-hesitance DM of two CFSs ${\mathbb {R}} _{1}$and ${\mathbb {R}} _{2},$denoted by $d_{EH}( {\mathbb {R}} _{1}\cup {\mathbb {R}} _{2},{\mathbb {R}} _{1}\cap {\mathbb {R}} _{2})$ or $d_{EH}( {\mathbb {R}} _{1}\cap {\mathbb {R}} _{2},{\mathbb {R}} _{1}\cup {\mathbb {R}} _{2})$,and is satisfied the function

$$\begin{aligned} d_{EH}( {\mathbb {R}} _{1}\cup {\mathbb {R}} _{2},{\mathbb {R}} _{1}\cap {\mathbb {R}} _{2})=\sqrt{\frac{ {\displaystyle \sum _{i=1}^{n}} \left[ \begin{array}{c} (\left( \ell _{ {\mathbb {R}} _{1}}\left( r_{i}\right) \vee \ell _{ {\mathbb {R}} _{2}}\left( r_{i}\right) \right) -\left( \ell _{ {\mathbb {R}} _{1}}\left( r_{i}\right) \wedge \ell _{ {\mathbb {R}} _{2}}\left( r_{i}\right) \right) )^{2}+\\ \frac{1}{2\pi ^{2}}(\left( w_{ {\mathbb {R}} _{1}}\left( r_{i}\right) \vee w_{ {\mathbb {R}} _{2}}\left( r_{i}\right) \right) -\left( w_{ {\mathbb {R}} _{1}}\left( r_{i}\right) \wedge w_{ {\mathbb {R}} _{2}}\left( r_{i}\right) \right) )^{2}+\\ (\left( \hslash _{ {\mathbb {R}} _{1}}\left( r_{i}\right) \vee \hslash _{ {\mathbb {R}} _{2}}\left( r_{i}\right) \right) -\left( \hslash _{ {\mathbb {R}} _{1}}\left( r_{i}\right) \wedge \hslash _{ {\mathbb {R}} _{2}}\left( r_{i}\right) \right) )^{2} \end{array} \right] }{3n}} \end{aligned}$$

and

$$\begin{aligned} d_{EH}( {\mathbb {R}} _{1}\cap {\mathbb {R}} _{2},{\mathbb {R}} _{1}\cup {\mathbb {R}} _{2})=\sqrt{\frac{ \begin{array}{c} {\displaystyle \sum _{i=1}^{n}} (\left( \ell _{ {\mathbb {R}} _{1}}\left( r_{i}\right) \wedge \ell _{ {\mathbb {R}} _{2}}\left( r_{i}\right) \right) -\left( \ell _{ {\mathbb {R}} _{1}}\left( r_{i}\right) \vee \ell _{ {\mathbb {R}} _{2}}\left( r_{i}\right) \right) )^{2}+\\ \frac{1}{2\pi ^{2}}(\left( w_{ {\mathbb {R}} _{1}}\left( r_{i}\right) \wedge w_{ {\mathbb {R}} _{2}}\left( r_{i}\right) \right) -\left( w_{ {\mathbb {R}} _{1}}\left( r_{i}\right) \vee w_{ {\mathbb {R}} _{2}}\left( r_{i}\right) \right) )^{2}+\\ (\left( \hslash _{ {\mathbb {R}} _{1}}\left( r_{i}\right) \wedge \hslash _{ {\mathbb {R}} _{2}}\left( r_{i}\right) \right) -\left( \hslash _{ {\mathbb {R}} _{1}}\left( r_{i}\right) \vee \hslash _{ {\mathbb {R}} _{2}}\left( r_{i}\right) \right) )^{2} \end{array} }{3n}}. \end{aligned}$$

Here $\wedge ,\vee$ are represents max-min-operators.

## Applications of complex fuzzy sets in decision-making problems

Various decision-making algorithms are designed for specific problems and scenarios. This section proposes a new decision-making method based on CFSs and CFDMs. CFS theory extends the capabilities of traditional fuzzy set theory by incorporating complex numbers, offering more nuanced representation and manipulation of uncertainty in decision-making problems. It gives decision-makers a powerful framework for dealing with complex and uncertain scenarios and making informed choices. In decision-making problems, CFSs can be used to more effectively model uncertain information, preferences, and conflicting criteria. The complex nature of the numbers allows decision-makers to consider a broader range of possibilities and make more informed choices. Moreover, as introduced in the paper, the algorithm likely provides a robust methodology for incorporating complex fuzzy distance measures into the decision-making process. This integration allows for a more nuanced consideration of uncertainty, imprecision, and complexity in real-world decision-making scenarios.

We introduce the following definitions that are relevant to our proposed decision-making algorithm.

### Definition 27

Let $\alpha _{i}=(\alpha _{1},\alpha _{2},\ldots ,\alpha _{i})$ be a collection of *i* alternatives and $c_{j}=(c_{1},c_{2},\ldots ,c_{j})$ be a collection of *j* attributes. Let $E=\{E_{1},E_{2},\ldots ,E_{k}\}$ be a set of *k* experts where $E_{k}$ denotes the *k*th expert who takes part in the decision making process. The attribute value of alternatives $\alpha _{i}$ for index $c_{j}$ is $C_{ij}=r_{C_{ij}}(c_{j})e^{iw_{C_{ij}}(c_{j})}$ which is the evaluated value provided by the expert $E_{k}$ for alternative $\alpha _{i}$ with respect to the criterion $c_{j}.$ So the table obtained from these evaluated values is:

**Table:** 

$E_{k}$	$\alpha _{i}$	$c_{1}$	$c_{2}$	.	.	.	$c_{j}$
	$\alpha _{1}$	$r_{C_{11}}(c_{1})e^{iw_{C_{11}}(c_{1})}$	$r_{C_{12}} (c_{2})e^{iw_{C_{12}}(c_{2})}$	.	.	.	$r_{C_{1j}}(c_{j})e^{iw_{C_{1j} }(c_{j})}$
$E_{1}$	$\alpha _{2}$	$r_{C_{21}}(c_{1})e^{iw_{C_{21}}(c_{1})}$	$r_{C_{22}}(c_{2})e^{iw_{C_{21}}(c_{2})}$	.	.	.	$r_{C_{2j}} (c_{j})e^{iw_{C_{2j}}(c_{j})}$
	...	...	...	.	.	.	...
	$\alpha _{i}$	$r_{C_{ij}}(c_{1})e^{iw_{C_{ij}}(c_{1})}$	$r_{C_{ij}} (c_{2})e^{iw_{C_{ij}}(c_{2})}$	.	.	.	$r_{C_{ij}}(c_{j})e^{iw_{C_{ij} }(c_{j})}$
...				.	.	.	...
	$\alpha _{1}$	$r_{C_{11}}(c_{1})e^{iw_{C_{11}}(c_{1})}$	$r_{C_{12}} (c_{2})e^{iw_{C_{12}}(c_{2})}$	.	.	.	$r_{C_{1j}}(c_{j})e^{iw_{C_{1j} }(c_{j})}$
$E_{2}$	$\alpha _{2}$	$r_{C_{21}}(c_{1})e^{iw_{C_{21}}(c_{1})}$	$r_{C_{22}}(c_{2})e^{iw_{C_{21}}(c_{2})}$	.	.	.	$r_{C_{2j}} (c_{j})e^{iw_{C_{2j}}(c_{j})}$
	...	...	...	.	.	.	...
	$\alpha _{i}$	$r_{C_{ij}}(c_{1})e^{iw_{C_{ij}}(c_{1})}$	$r_{C_{ij}} (c_{2})e^{iw_{C_{ij}}(c_{2})}$	.	.	.	$r_{C_{ij}}(c_{j})e^{iw_{C_{ij} }(c_{j})}$
...				.	.	.	
	$\alpha _{1}$	$r_{C_{11}}(c_{1})e^{iw_{C_{11}}(c_{1})}$	$r_{C_{12}} (c_{2})e^{iw_{C_{12}}(c_{2})}$	.	.	.	$r_{C_{1j}}(c_{j})e^{iw_{C_{1j} }(c_{j})}$
$E_{k}$	$\alpha _{2}$	$r_{C_{21}}(c_{1})e^{iw_{C_{21}}(c_{1})}$	$r_{C_{22}}(c_{2})e^{iw_{C_{21}}(c_{2})}$	.	.	.	$r_{C_{2j}} (c_{j})e^{iw_{C_{2j}}(c_{j})}$
	...	...	...	.	.	.	...
	$\alpha _{i}$	$r_{C_{ij}}(c_{1})e^{iw_{C_{ij}}(c_{1})}$	$r_{C_{ij}} (c_{2})e^{iw_{C_{ij}}(c_{2})}$	.	.	.	$r_{C_{ij}}(c_{j})e^{iw_{C_{ij} }(c_{j})}$

### Definition 28

Let $\alpha _{i}=(\alpha _{1},\alpha _{2},\ldots ,\alpha _{i})$ be a collection of *i* alternatives and $c_{j}=(c_{1},c_{2},\ldots ,c_{j})$ be a collection of *j* attributes. Let $E=\{E_{1},E_{2},\ldots ,E_{k}\}$ be a set of *k* experts where $E_{k}$ denotes the *k*th expert who takes part in the decision making process. The attribute value of alternatives $\alpha _{i}$ for index $c_{j}$ is $C_{ij}=r_{C_{ij}}(c_{j})e^{iw_{C_{ij}}(c_{j})}$ which is the evaluated value provided by the expert $E_{k}$ for alternative $\alpha _{i}$ with respect to the criterion $c_{j}.$ Then, the entries of the distance matrix are denoted by $d(\alpha _{i}^{(E_{l})},\alpha _{i}^{(E_{k})})$ and is defined by Table 1:

**Table 1:** Evaluated values in the environment of complex fuzzy sets.

$$\left[ D_{M}\right] _{3\times 3}= \begin{bmatrix} d(\alpha _{1}^{(E_{1})},\alpha _{1}^{(E_{2})}) &{} d(\alpha _{1}^{(E_{1})} ,\alpha _{1}^{(E_{3})}) &{} . &{} . &{} . &{} d(\alpha _{1}^{(E_{k-1})},\alpha _{1}^{(E_{k})})\\ d(\alpha _{2}^{(E_{1})},\alpha _{2}^{(E_{2})}) &{} d(\alpha _{2}^{(E_{1})} ,\alpha _{2}^{(E_{3})}) &{} . &{} . &{} . &{} d(\alpha _{2}^{(E_{k-1})},\alpha _{2}^{(E_{k})})\\ . &{} . &{} . &{} . &{} . &{} .\\ . &{} . &{} . &{} . &{} . &{} .\\ . &{} . &{} . &{} . &{} . &{} .\\ d(\alpha _{i}^{(E_{1})},\alpha _{i}^{(E_{2})}) &{} d(\alpha _{i}^{(E_{1})} ,\alpha _{i}^{(E_{3})}) &{} . &{} . &{} . &{} d(\alpha _{i}^{(E_{k-1})},\alpha _{i}^{(E_{k})}) \end{bmatrix}$$	

Where $d(\alpha _{i}^{(E_{l})},\alpha _{i}^{(E_{k})})$ denotes the proposed Hesitance or Euclidean CFDMs.

### Definition 29

The score function $S(\alpha _{i})$ of alternative $\alpha _{i};$ $i=1,2,\ldots ,n$ is defined as

$$\begin{aligned} S(\alpha _{i})=\frac{\overset{n}{\underset{i=1}{\sum }}d(\alpha _{i}^{(E_{j} )},\alpha _{i}^{(E_{k})})}{n}, \end{aligned}$$

where $d(\alpha _{i}^{(E_{j})},\alpha _{i}^{(E_{k})})$ denotes the Hesitance or Euclidean CFDMs.

### Decision-making algorithm

The general outline of steps involved in the proposed decision-making algorithms are given below. These steps are based on CFDMs, distance matrix, score functions, and rank of alternatives.

**Step 1.**

Construct the decision-making table, defined in (27), in the environment of CFSs.

**Step 2.**

Compute the Hesitance CFDM or Euclidean CFDM between the values evaluated by different experts corresponding to alternatives $\alpha _{i}.$

**Step 3.**

Construct the distance matrix, defined in (28), based on the values of Hesitance CFDMs or Euclidean CFDMs.

**Step 4.**

Calculate the score functions $S(\alpha _{i}),$ defined in (29), corresponding to each alternatives $\alpha _{i}.$

**Step 5.**

Rank the alternatives based on the score functions and, hence, select the most desirable one(s).

### Case study

In this subsection, we utilized the proposed CFDMs to choose the most desirable online business.

Running a successful online business involves monitoring various parameters to ensure that your operations are efficient, your products are well-received, and your overall business is thriving. Here are some key parameters to consider:

1. Sales Performance

*Sales Volume.* Monitor your overall sales to gauge the success of your business.

*Sales by Product.* Understand which products are performing well and which may need attention.

2. Customer Metrics

*Customer Reviews.* Positive reviews can boost your product’s visibility and credibility.

*Customer Feedback.* Pay attention to customer feedback to identify areas for improvement.

3. Inventory Management

*Inventory Levels.* Ensure that you maintain adequate stock to meet demand.

*Inventory Turnover.* Aim for a balance between keeping enough stock and avoiding excess inventory.

4. Financial Metrics

*Profit Margins.* Analyze your profit margins on each product to ensure profitability.

*Return on Investment (ROI).* Evaluate the effectiveness of your investments in inventory and advertising.

5. Advertising and Marketing

*Ad Performance.* Monitor the effectiveness of your Amazon advertising campaigns.

*Conversion Rates.* Track how well your product listings convert views into sales.

Regularly reviewing and adjusting these parameters will help you make informed decisions, optimize your operations and ensure the long-term success of your online business.

### Problem description

Let $L=\{L_{1}=eBay,L_{2}=Alibaba,L_{3}=Amazone\}$ be a collection of three online business, $C=\{C_{1},C_{2},C_{3},C_{4},C_{5}\}$ be a collection of five attributes where $C_{1},$ $C_{2},$ $C_{3},$ $C_{4},$ $C_{5}$ represent sales performance, customer metrics, inventory management, financial metrics, and advertising and marketing, respectively. These are the common attributes when choosing an online business.

Step 1. Let $E=\{E_{1},E_{2},E_{3}\}$ be a set of three experts who take part in the decision-making process. Assume that Mr. Salar wants to start an online business under a decision-making problem. To conduct the assessment and selection procedure, specifically to review alternatives based on five decision criteria, Mr. Salar designates three decision-making specialists, $E=\{E_{1},E_{2},E_{3}\}$. By engaging in discussions with the three experts, five qualitative criteria about online business are considered. According to the expertise of the three experts, the complex fuzzy information about online business is given in the following Table 2.

**Table 2:** Evaluated values of alternatives.

$E_{k}$	$L_{i}$	$c_{1}$	$c_{2}$	$c_{3}$	$c_{4}$	$c_{5}$
$E_{1}$	$L_{1}$	$0.3e^{0.8\pi }$	$0.8e^{0.4\pi }$	$0.3e^{0.2\pi }$	$0.4e^{1.8\pi }$	$0.9e^{1\pi }$
	$L_{2}$	$0.1e^{0.2\pi }$	$0.5e^{1.2\pi }$	$0e^{2\pi }$	$0.7e^{1.4\pi }$	$0.4e^{0.6\pi }$
	$L_{3}$	$0.3e^{0.5\pi }$	$0.8e^{\pi }$	$0.1e^{1.6\pi }$	$0.9e^{1.2\pi }$	$0.5e^{0.9\pi }$
$E_{2}$	$L_{1}$	$0.6e^{0.4\pi }$	$0.1e^{1.1\pi }$	$0.9e^{1.5\pi }$	$0.2e^{2\pi }$	$0.7e^{0.6\pi }$
	$L_{2}$	$0.4e^{0.1\pi }$	$0.5e^{\pi }$	$0.3e^{2\pi }$	$1e^{1.8\pi }$	$0.6e^{0.8\pi }$
	$L_{3}$	$1e^{1\pi }$	$0e^{2\pi }$	$0.7e^{0.2\pi }$	$0.2e^{0.6\pi }$	$0.9e^{1.4\pi }$
$E_{3}$	$L_{1}$	$0e^{0.5\pi }$	$1e^{1\pi }$	$0.6e^{0.7\pi }$	$0.3e^{2\pi }$	$0.8e^{1.6\pi }$
	$L_{2}$	$0.5e^{0.6\pi }$	$0.7e^{1.3\pi }$	$0.9e^{1.6\pi }$	$0e^{1.4\pi }$	$0.2e^{2\pi }$
	$L_{3}$	$1e^{0.8\pi }$	$0.5e^{0.4\pi }$	$0.7e^{1.5\pi }$	$0.6e^{1\pi }$	$0.1e^{0.7\pi }$

Step 2. Calculate the Hesitance CFDM between the values evaluated by different experts corresponding to alternatives $L_{i};$ $i=1,2,3.$

$$\begin{aligned} d(L_{1}^{(E_{1})},L_{1}^{(E_{2})})&=\frac{\left[ \begin{array}{c} \left( |0.3-0.6|+\frac{1}{2\pi }|0.8\pi -0.4\pi |+|0.1-0.4|\right) +\\ \left( |0.8-0.1|+\frac{1}{2\pi }|0.4\pi -1.1\pi |+|0.6-0.45|\right) +\\ \left( |0.3-0.9|+\frac{1}{2\pi }|0.2\pi -1.5\pi |+|0.2-0.25|\right) +\\ \left( |0.4-0.2|+\frac{1}{2\pi }|1.8\pi -2\pi |+|0.5-0.8|\right) +\\ \left( |0.9-0.7|+\frac{1}{2\pi }|\pi -0.6\pi |+|0.4-0.4|\right) \end{array} \right] }{3(5)},\\&=\frac{\left[ \begin{array}{c} 0.3+0.2+0.3+0.7+0.35+0.15+0.6+0.65\\ +0.05+0.2+0.1+03+0.2+0.2+0.05 \end{array} \right] }{15},\\&=0.29.\\ d(L_{1}^{(E_{1})},L_{1}^{(E_{3})})&=\frac{\left[ \begin{array}{c} \left( |0.3-0|+\frac{1}{2\pi }|0.8\pi -0.5\pi |+|0.4-0.5|\right) +\\ \left( |0.8-1|+\frac{1}{2\pi }|0.4\pi -1\pi |+|0.6-0.5|\right) +\\ \left( |0.3-0.6|+\frac{1}{2\pi }|0.2\pi -0.7\pi |+|0.2-0|\right) +\\ \left( |0.4-0.3|+\frac{1}{2\pi }|1.8\pi -2\pi |+|0.5-0.7|\right) +\\ \left( |0.9-0.8|+\frac{1}{2\pi }|1\pi -1.6\pi |+|0.4-0|\right) \end{array} \right] }{3(5)},\\&=\frac{\left[ \begin{array}{c} 0.3+0.15+0.15+0.2+0.3+0.1+0.3+\\ 0.25+0.2+0.1+0.6+0.2+0.1+0.3+0.4 \end{array} \right] }{15},\\&=0.24. \\ d(L_{1}^{(E_{2})},L_{1}^{(E_{3})})&=\frac{\left[ \begin{array}{c} \left( |0.6-0|+\frac{1}{2\pi }|0.4\pi -0.5\pi |+|0.4-0.25|\right) +\\ \left( |0.1-1|+\frac{1}{2\pi }|1.1\pi -\pi |+|0.45-0.5|\right) +\\ \left( |0.9-0.6|+\frac{1}{2\pi }|1.5\pi -0.7\pi |+|0.15-0.25|\right) +\\ \left( |0.2-0.3|+\frac{1}{2\pi }|2\pi -2\pi |+|0.8-0.7|\right) +\\ \left( |0.7-0.8|+\frac{1}{2\pi }|0.6\pi -1.6\pi |+|0.4-0|\right) \end{array} \right] }{3(5)},\\&=\frac{\left[ \begin{array}{c} 0.5+0.05+0.15+0.9+0.05+0.05+0.3+0.4\\ +0.1+0.1+0+0.1+0.1+0.5+0.4 \end{array} \right] }{15},\\&=0.25 \end{aligned}$$

Similarly,

$$\begin{aligned} d(L_{2}^{(E_{1})},L_{2}^{(E_{2})})&=0.17,\\ d(L_{2}^{(E_{1})},L_{2}^{(E_{3})})&=0.4,\\ d(L_{2}^{(E_{2})},L_{2}^{(E_{3})})&=0.38. \\ d(L_{3}^{(E_{1})},L_{3}^{(E_{2})})&=0.41,\\ d(L_{3}^{(E_{1})},L_{3}^{(E_{3})})&=0.29,\\ d(L_{3}^{(E_{2})},L_{3}^{(E_{3})})&=0.36. \end{aligned}$$

Now, the distance matrix based on the values of Hesitance CFDMs is given below:

$$\begin{aligned} \left[ D_{M}\right] _{3\times 3}&= \begin{bmatrix} d(L_{1}^{(E_{1})},L_{1}^{(E_{2})}) &{} d(L_{1}^{(E_{1})},L_{1}^{(E_{3})}) &{} d(L_{1}^{(E_{2})},L_{1}^{(E_{3})})\\ d(L_{2}^{(E_{1})},L_{2}^{(E_{2})}) &{} d(L_{2}^{(E_{1})},L_{2}^{(E_{3})}) &{} d(L_{2}^{(E_{2})},L_{2}^{(E_{3})})\\ d(L_{3}^{(E_{1})},L_{3}^{(E_{2})}) &{} d(L_{3}^{(E_{1})},L_{3}^{(E_{3})}) &{} d(L_{3}^{(E_{2})},L_{3}^{(E_{3})}) \end{bmatrix} ,\\&= \begin{bmatrix} 0.29 &{} 0.24 &{} 0.25\\ 0.17 &{} 0.4 &{} 0.38\\ 0.4 &{} 0.29 &{} 0.36 \end{bmatrix} . \end{aligned}$$

The score functions $S(L_{1}),$ $S(L_{2}),$ $S(L_{3})$ of each alternatives $L_{1},$ $L_{2}$ and $L_{3}$ are computed as:

$$\begin{aligned} S(L_{i})&=\frac{\overset{3}{\underset{i=1}{\sum }}d(L_{i}^{(E_{j})} ,L_{i}^{(E_{k})})}{3},\\ S(L_{1})&=\frac{0.29+0.24+0.25}{3},\\&=0.26,\\ S(L_{2})&=\frac{0.17+0.4+0.38}{3},\\&=0.32,\\ S(L_{3})&=\frac{0.4+0.29+0.36}{3},\\&=0.35. \end{aligned}$$

The rank of the alternatives based on the score functions is:

$$\begin{aligned} S(L_{1})<S(L_{2})<S(L_{3}) \end{aligned}$$

As a result, the score functions based on Hesitance CFDMs choose Amazon $(L_{3})$ as the best platform for online business.

### Euclidean hesitance complex fuzzy distance measures

Now, we revise the decision-making algorithm in the environment of Euclidean Hesitance CFDMs. The computed values of the Euclidean Hesitance CFDMs are given below:

$$\begin{aligned} d(L_{1}^{(E_{1})},L_{1}^{(E_{2})})= & {} \left( \frac{ \begin{array}{c} \left( \left( 0.3-0.6\right) ^{2}+\frac{1}{2\pi ^{2}}\left( 0.8\pi -0.4\pi \right) ^{2}+\left( 0.1-0.4\right) ^{2}\right) +\\ \left( \left( 0.8-0.1\right) ^{2}+\frac{1}{2\pi ^{2}}\left( 0.4\pi -1.1\pi \right) ^{2}+\left( 0.6-0.45\right) ^{2}\right) +\\ \left( \left( 0.3-0.9\right) ^{2}+\frac{1}{2\pi ^{2}}\left( 0.2\pi -1.5\pi \right) ^{2}+\left( 0.2-0.25\right) ^{2}\right) +\\ \left( \left( 0.4-0.2\right) ^{2}+\frac{1}{2\pi ^{2}}\left( 1.8\pi -2\pi \right) ^{2}+\left( 0.5-0.8\right) ^{2}\right) +\\ \left( \left( 0.9-0.7\right) ^{2}+\frac{1}{2\pi ^{2}}\left( \pi -0.6\pi \right) ^{2}+\left( 0.4-0.4\right) ^{2}\right) \end{array} }{3(5)}\right) ^{\frac{1}{2}}, \\ d(L_{1}^{(E_{1})},L_{1}^{(E_{2})})= & {} \left[ \frac{ \begin{array}{c} 0.09+0.04+0.09+0.49+0.125+0.03+0.36+0.42\\ +0.01+0.04+0.01+0.09+0.04+0.04+0.01 \end{array} }{15}\right] ^{\frac{1}{2}},\\&=\left[ \frac{1.86}{15}\right] ^{\frac{1}{2}},\\&=0.4. \\ d(L_{1}^{(E_{1})},L_{1}^{(E_{3})})= & {} \left[ \frac{ \begin{array}{c} \left( \left( 0.3-0\right) ^{2}+\frac{1}{2\pi ^{2}}\left( 0.8\pi -0.5\pi \right) ^{2}+\left( 0.4-0.5\right) ^{2}\right) +\\ \left( \left( 0.8-1\right) ^{2}+\frac{1}{2\pi ^{2}}\left( 0.4\pi -1\pi \right) ^{2}+\left( 0.6-0.5\right) ^{2}\right) +\\ \left( \left( 0.3-0.6\right) ^{2}+\frac{1}{2\pi ^{2}}\left( 0.2\pi -0.7\pi \right) ^{2}+\left( 0.2-0\right) ^{2}\right) +\\ \left( \left( 0.4-0.3\right) ^{2}+\frac{1}{2\pi ^{2}}\left( 1.8\pi -2\pi \right) ^{2}+\left( 0.5-0.7\right) ^{2}\right) +\\ \left( \left( 0.9-0.8\right) ^{2}+\frac{1}{2\pi ^{2}}\left( 1\pi -1.6\pi \right) ^{2}+\left( 0.4-0\right) ^{2}\right) \end{array}}{3(5)}\right] ^{\frac{1}{2}}\\ d(L_{1}^{(E_{1})},L_{1}^{(E_{3})})= & {} \left[ \frac{ \begin{array}{c} 0.09+0.03+0.03+0.04+0.09+0.01+0.09+0.06\\ +0.04+0.01+0.36+0.04+0.01+0.09+0.16 \end{array}}{15}\right] ^{\frac{1}{2}},\\= & {} \left[ \frac{1.08}{15}\right] ^{\frac{1}{2}},\\= & {} 0.27.\\ d(L_{1}^{(E_{2})},L_{1}^{(E_{3})})= & {} \left[ \frac{ \begin{array}{c} \left( \left( 0.6-0\right) ^{2}+\frac{1}{2\pi ^{2}}\left( 0.4\pi -0.5\pi \right) ^{2}+\left( 0.4-0.25\right) ^{2}\right) +\\ \left( \left( 0.1-1\right) ^{2}+\frac{1}{2\pi ^{2}}\left( 1.1\pi -\pi \right) ^{2}+\left( 0.45-0.5\right) ^{2}\right) +\\ \left( \left( 0.9-0.6\right) ^{2}+\frac{1}{2\pi ^{2}}\left( 1.5\pi -0.7\pi \right) ^{2}+\left( 0.15-0.25\right) ^{2}\right) +\\ \left( \left( 0.2-0.3\right) ^{2}+\frac{1}{2\pi ^{2}}\left( 2\pi -2\pi \right) ^{2}+\left( 0.8-0.7\right) ^{2}\right) +\\ \left( \left( 0.7-0.8\right) ^{2}+\frac{1}{2\pi ^{2}}\left( 0.6\pi -1.6\pi \right) ^{2}+\left( 0.4-0\right) ^{2}\right) \end{array}}{3(5)}\right] ^{\frac{1}{2}},\\ d(L_{1}^{(E_{2})},L_{1}^{(E_{3})})= & {} \left[ \frac{ \begin{array}{c} 0.36+0.01+0.03+0.81+0.01+0.01+0.09+0.16\\ +0.01+0.01+0+0.01+0.01+0.25+0.16 \end{array} }{15}\right] ^{\frac{1}{2}},\\= & {} 0.36. \end{aligned}$$

Similarly,

$$\begin{aligned} d(L_{2}^{(E_{1})},L_{2}^{(E_{2})})&=0.2,\\ d(L_{2}^{(E_{1})},L_{2}^{(E_{3})})&=0.51,\\ d(L_{2}^{(E_{2})},L_{2}^{(E_{3})})&=0.46. \\ d(L_{3}^{(E_{1})},L_{3}^{(E_{2})})&=0.47,\\ d(L_{3}^{(E_{1})},L_{3}^{(E_{3})})&=0.37,\\ d(L_{3}^{(E_{2})},L_{3}^{(E_{3})})&=0.46. \end{aligned}$$

Now, the distance matrix based on the values of Euclidean Hesitance CFDMs is given below:

$$\begin{aligned} \left[ D_{M}\right] _{3\times 3}&= \begin{bmatrix} d(L_{1}^{(E_{1})},L_{1}^{(E_{2})}) &{} d(L_{1}^{(E_{1})},L_{1}^{(E_{3})}) &{} d(L_{1}^{(E_{2})},L_{1}^{(E_{3})})\\ d(L_{2}^{(E_{1})},L_{2}^{(E_{2})}) &{} d(L_{2}^{(E_{1})},L_{2}^{(E_{3})}) &{} d(L_{2}^{(E_{2})},L_{2}^{(E_{3})})\\ d(L_{3}^{(E_{1})},L_{3}^{(E_{2})}) &{} d(L_{3}^{(E_{1})},L_{3}^{(E_{3})}) &{} d(L_{3}^{(E_{2})},L_{3}^{(E_{3})}) \end{bmatrix} ,\\&= \begin{bmatrix} 0.4 &{} 0.27 &{} 0.36\\ 0.2 &{} 0.51 &{} 0.46\\ 0.47 &{} 0.37 &{} 0.46 \end{bmatrix} . \end{aligned}$$

The score functions $S(L_{1}),$ $S(L_{2}),$ $S(L_{3})$ of each alternatives $L_{1},$ $L_{2}$ and $L_{3}$ are computed as:

$$\begin{aligned} S(L_{i})&=\frac{\overset{3}{\underset{i=1}{\sum }}d(L_{i}^{(E_{j})} ,L_{i}^{(E_{k})})}{3},\\ S(L_{1})&=\frac{0.4+0.27+0.36}{3},\\&=0.34,\\ S(L_{2})&=\frac{0.2+0.51+0.46}{3},\\&=0.39,\\ S(L_{3})&=\frac{0.47+0.37+0.46}{3},\\&=0.43. \end{aligned}$$

The rank of the alternatives based on the score functions is

$$\begin{aligned} S(L_{1})<S(L_{2})<S(L_{3}) \end{aligned}$$

As a result, the score functions based on Euclidean Hesitance CFDMs choose Amazon $(L_{3})$ as the best choice.

## Comparison analysis

In this section, we discussed the comparison of the CFHDM and CFEHDM with the CFNHDM^40^ and complex fuzzy Normalized Euclidean DM^40^. If we take some restrictions on CFHDM and CFEHDM with the CFNHDM, then the proposed CFDMs are reduced to CFNHDM and complex fuzzy Normalized Euclidean DM. In remarks 1-2, the comparison is established. We have also discussed the decision-making problems based on other CFDM without hesitancy degree. The comparative study is given below:

### Remark 1

The CFHDM is reduced to CFNHDM if we consider the Hesitancy degree as zero.

40 $$\begin{aligned} d_{H}\left( {\mathbb {R}} _{1},{\mathbb {R}} _{2}\right) =\frac{ {\displaystyle \sum _{i=1}^{n}} \left[ \mid \ell _{ {\mathbb {R}} _{1}}\left( r_{i}\right) -\ell _{ {\mathbb {R}} _{2}}\left( r_{i}\right) \mid +\frac{1}{2\pi }\mid w_{ {\mathbb {R}} _{1}}\left( r_{i}\right) -w_{ {\mathbb {R}} _{2}}\left( r_{i}\right) \mid \right] }{2n}. \end{aligned}$$

### Remark 2

The CFEHDM reduces to complex fuzzy Normalized Euclidean DM if we considered the Hesitancy degree as zero.

41 $$\begin{aligned} d_{EH}\left( {\mathbb {R}} _{1},{\mathbb {R}} _{2}\right) =\sqrt{\frac{ {\displaystyle \sum _{i=1}^{n}} \left[ (\ell _{ {\mathbb {R}} _{1}}\left( r_{i}\right) -\ell _{ {\mathbb {R}} _{2}}\left( r_{i}\right) )^{2}+\frac{1}{2\pi ^{2}}(w_{ {\mathbb {R}} _{1}}\left( r_{i}\right) -w_{ {\mathbb {R}} _{2}}\left( r_{i}\right) )^{2}\right] }{2n}}. \end{aligned}$$

The above decision-making problem can be solved using the CFNHDM proposed in Remark 1. the CFNHDM between the values evaluated by different experts corresponding to alternatives $L_{i};$ $i=1,2,3.$

$$\begin{aligned} d(L_{1}^{(E_{1})},L_{1}^{(E_{2})})&=\frac{\left[ \begin{array}{c} \left( |0.3-0.6|+\frac{1}{2\pi }|0.8\pi -0.4\pi |\right) +\\ \left( |0.8-0.1|+\frac{1}{2\pi }|0.4\pi -1.1\pi |\right) +\\ \left( |0.3-0.9|+\frac{1}{2\pi }|0.2\pi -1.5\pi |\right) +\\ \left( |0.4-0.2|+\frac{1}{2\pi }|1.8\pi -2\pi |\right) +\\ \left( |0.9-0.7|+\frac{1}{2\pi }|\pi -0.6\pi |\right) \end{array} \right] }{2(5)},\\&=\frac{\left[ \begin{array}{c} 0.3+0.2+0.7+0.35+0.6+0.65\\ +0.2+0.1+0.2+0.2 \end{array} \right] }{10},\\&=0.35. \\ d(L_{1}^{(E_{1})},L_{1}^{(E_{3})})&=\frac{\left[ \begin{array}{c} \left( |0.3-0|+\frac{1}{2\pi }|0.8\pi -0.5\pi |\right) +\\ \left( |0.8-1|+\frac{1}{2\pi }|0.4\pi -1\pi |\right) +\\ \left( |0.3-0.6|+\frac{1}{2\pi }|0.2\pi -0.7\pi |\right) +\\ \left( |0.4-0.3|+\frac{1}{2\pi }|1.8\pi -2\pi |\right) +\\ \left( |0.9-0.8|+\frac{1}{2\pi }|1\pi -1.6\pi |\right) \end{array} \right] }{2(5)},\\&=\frac{\left[ \begin{array}{c} 0.3+0.15+0.2+0.3+0.3+\\ 0.25+0.1+0.6+0.1+0.3+ \end{array} \right] }{10},\\&=0.26. \\ d(L_{1}^{(E_{2})},L_{1}^{(E_{3})})&=\frac{\left[ \begin{array}{c} \left( |0.6-0|+\frac{1}{2\pi }|0.4\pi -0.5\pi |\right) +\\ \left( |0.1-1|+\frac{1}{2\pi }|1.1\pi -\pi |\right) +\\ \left( |0.9-0.6|+\frac{1}{2\pi }|1.5\pi -0.7\pi |\right) +\\ \left( |0.2-0.3|+\frac{1}{2\pi }|2\pi -2\pi |\right) +\\ \left( |0.7-0.8|+\frac{1}{2\pi }|0.6\pi -1.6\pi |\right) \end{array} \right] }{2(5)},\\&=\frac{\left[ \begin{array}{c} 0.5+0.05+0.9+0.05+0.3+0.4\\ +0.1+0+0.1+0.5 \end{array} \right] }{10},\\&=0.29. \end{aligned}$$

Similarly,

$$\begin{aligned} d(L_{2}^{(E_{1})},L_{2}^{(E_{2})})&=0.17,\\ d(L_{2}^{(E_{1})},L_{2}^{(E_{3})})&=0.48,\\ d(L_{2}^{(E_{2})},L_{2}^{(E_{3})})&=0.37. \\ d(L_{3}^{(E_{1})},L_{3}^{(E_{2})})&=0.52,\\ d(L_{3}^{(E_{1})},L_{3}^{(E_{3})})&=0.3,\\ d(L_{3}^{(E_{2})},L_{3}^{(E_{3})})&=0.38. \end{aligned}$$

Now, the distance matrix based on the values of CFNHDMs is given below

$$\begin{aligned} \left[ D_{M}\right] _{3\times 3}&= \begin{bmatrix} d(L_{1}^{(E_{1})},L_{1}^{(E_{2})}) &{} d(L_{1}^{(E_{1})},L_{1}^{(E_{3})}) &{} d(L_{1}^{(E_{2})},L_{1}^{(E_{3})})\\ d(L_{2}^{(E_{1})},L_{2}^{(E_{2})}) &{} d(L_{2}^{(E_{1})},L_{2}^{(E_{3})}) &{} d(L_{2}^{(E_{2})},L_{2}^{(E_{3})})\\ d(L_{3}^{(E_{1})},L_{3}^{(E_{2})}) &{} d(L_{3}^{(E_{1})},L_{3}^{(E_{3})}) &{} d(L_{3}^{(E_{2})},L_{3}^{(E_{3})}) \end{bmatrix} ,\\&= \begin{bmatrix} 0.35 &{} 0.26 &{} 0.29\\ 0.17 &{} 0.48 &{} 0.37\\ 0.52 &{} 0.3 &{} 0.38 \end{bmatrix} . \end{aligned}$$

The score functions $S(L_{1}),$ $S(L_{2}),$ $S(L_{3})$ of each alternatives $L_{1},$ $L_{2}$ and $L_{3}$ are computed as:

$$\begin{aligned} S(L_{i})&=\frac{\overset{3}{\underset{i=1}{\sum }}d(L_{i}^{(E_{j})} ,L_{i}^{(E_{k})})}{3},\\ S(L_{1})&=\frac{0.35+0.26+0.29}{3},\\&=0.29,\\ S(L_{2})&=\frac{0.17+0.48+0.37}{3},\\&=0.34,\\ S(L_{3})&=\frac{0.52+0.3+0.38}{3},\\&=0.4. \end{aligned}$$

The rank of the alternatives based on the score functions is

$$\begin{aligned} S(L_{1})<S(L_{2})<S(L_{3}) \end{aligned}$$

As a result, the score functions based on CFNHDMs choose the Amazone $(L_{3})$ as the best choice.

### Complex fuzzy normalized Euclidean distance measures

Now, we revise the decision-making algorithm in the complex fuzzy Normalized Euclidean DMs environment. The computed values of the complex fuzzy Normalized Euclidean DMs are given below:

$$\begin{aligned} d(L_{1}^{(E_{1})},L_{1}^{(E_{2})})&=\left( \frac{ \begin{array}{c} \left( \left( 0.3-0.6\right) ^{2}+\frac{1}{2\pi ^{2}}\left( 0.8\pi -0.4\pi \right) ^{2}\right) +\\ \left( \left( 0.8-0.1\right) ^{2}+\frac{1}{2\pi ^{2}}\left( 0.4\pi -1.1\pi \right) ^{2}\right) +\\ \left( \left( 0.3-0.9\right) ^{2}+\frac{1}{2\pi ^{2}}\left( 0.2\pi -1.5\pi \right) ^{2}\right) +\\ \left( \left( 0.4-0.2\right) ^{2}+\frac{1}{2\pi ^{2}}\left( 1.8\pi -2\pi \right) ^{2}\right) +\\ \left( \left( 0.9-0.7\right) ^{2}+\frac{1}{2\pi ^{2}}\left( \pi -0.6\pi \right) ^{2}\right) \end{array}}{2(5)}\right) ^{\frac{1}{2}},\\ d(L_{1}^{(E_{1})},L_{1}^{(E_{2})})&=\left[ \frac{ \begin{array}{c} 0.09+0.04+0.49+0.125+0.36+0.42\\ +0.04+0.01+0.04+0.04 \end{array}}{10}\right] ^{\frac{1}{2}},\\&=\left[ \frac{1.655}{10}\right] ^{\frac{1}{2}},\\&=0.41.\\ d(L_{1}^{(E_{1})},L_{1}^{(E_{3})})&=\left[ \frac{ \begin{array}{c} \left( \left( 0.3-0\right) ^{2}+\frac{1}{2\pi ^{2}}\left( 0.8\pi -0.5\pi \right) ^{2}\right) +\\ \left( \left( 0.8-1\right) ^{2}+\frac{1}{2\pi ^{2}}\left( 0.4\pi -1\pi \right) ^{2}\right) +\\ \left( \left( 0.3-0.6\right) ^{2}+\frac{1}{2\pi ^{2}}\left( 0.2\pi -0.7\pi \right) ^{2}\right) +\\ \left( \left( 0.4-0.3\right) ^{2}+\frac{1}{2\pi ^{2}}\left( 1.8\pi -2\pi \right) ^{2}\right) +\\ \left( \left( 0.9-0.8\right) ^{2}+\frac{1}{2\pi ^{2}}\left( 1\pi -1.6\pi \right) ^{2}\right) \end{array} }{2(5)}\right] ^{\frac{1}{2}}, \\ d(L_{1}^{(E_{1})},L_{1}^{(E_{3})})&=\left[ \frac{ \begin{array}{c} 0.09+0.03+0.04+0.09+0.09+0.06\\ +0.01+0.36+0.01+0.09 \end{array} }{10}\right] ^{\frac{1}{2}},\\&=\left[ \frac{0.87}{10}\right] ^{\frac{1}{2}},\\&=0.29. \\ d(L_{1}^{(E_{2})},L_{1}^{(E_{3})})&=\left[ \frac{ \begin{array}{c} \left( \left( 0.6-0\right) ^{2}+\frac{1}{2\pi ^{2}}\left( 0.4\pi -0.5\pi \right) ^{2}\right) +\\ \left( \left( 0.1-1\right) ^{2}+\frac{1}{2\pi ^{2}}\left( 1.1\pi -\pi \right) ^{2}\right) +\\ \left( \left( 0.9-0.6\right) ^{2}+\frac{1}{2\pi ^{2}}\left( 1.5\pi -0.7\pi \right) ^{2}\right) +\\ \left( \left( 0.2-0.3\right) ^{2}+\frac{1}{2\pi ^{2}}\left( 2\pi -2\pi \right) ^{2}\right) +\\ \left( \left( 0.7-0.8\right) ^{2}+\frac{1}{2\pi ^{2}}\left( 0.6\pi -1.6\pi \right) ^{2}\right) \end{array} }{2(5)}\right] ^{\frac{1}{2}}, \\ d(L_{1}^{(E_{2})},L_{1}^{(E_{3})})&=\left[ \frac{ \begin{array}{c} 0.36+0.01+0.81+0.01+0.09+0.16\\ +0.01+0+0.01+0.25 \end{array} }{10}\right] ^{\frac{1}{2}},\\&=0.41. \end{aligned}$$

Similarly,

$$\begin{aligned} d(L_{2}^{(E_{1})},L_{2}^{(E_{2})})&=0.2,\\ d(L_{2}^{(E_{1})},L_{2}^{(E_{3})})&=0.51,\\ d(L_{2}^{(E_{2})},L_{2}^{(E_{3})})&=0.46. \\ d(L_{3}^{(E_{1})},L_{3}^{(E_{2})})&=0.47,\\ d(L_{3}^{(E_{1})},L_{3}^{(E_{3})})&=0.37,\\ d(L_{3}^{(E_{2})},L_{3}^{(E_{3})})&=0.46. \end{aligned}$$

Now, the distance matrix based on the values of complex fuzzy Normalized Euclidean DMs is given below:

$$\begin{aligned} \left[ D_{M}\right] _{3\times 3}&= \begin{bmatrix} d(L_{1}^{(E_{1})},L_{1}^{(E_{2})}) &{} d(L_{1}^{(E_{1})},L_{1}^{(E_{3})}) &{} d(L_{1}^{(E_{2})},L_{1}^{(E_{3})})\\ d(L_{2}^{(E_{1})},L_{2}^{(E_{2})}) &{} d(L_{2}^{(E_{1})},L_{2}^{(E_{3})}) &{} d(L_{2}^{(E_{2})},L_{2}^{(E_{3})})\\ d(L_{3}^{(E_{1})},L_{3}^{(E_{2})}) &{} d(L_{3}^{(E_{1})},L_{3}^{(E_{3})}) &{} d(L_{3}^{(E_{2})},L_{3}^{(E_{3})}) \end{bmatrix} ,\\&= \begin{bmatrix} 0.4 &{} 0.27 &{} 0.36\\ 0.2 &{} 0.6 &{} 0.45\\ 0.55 &{} 0.36 &{} 0.47 \end{bmatrix} . \end{aligned}$$

The score functions $S(L_{1}),$ $S(L_{2}),$ $S(L_{3})$ of each alternatives $L_{1},$ $L_{2}$ and $L_{3}$ are computed as:

$$\begin{aligned} S(L_{i})&=\frac{\overset{3}{\underset{i=1}{\sum }}d(L_{i}^{(E_{j})} ,L_{i}^{(E_{k})})}{3},\\ S(L_{1})&=\frac{0.4+0.27+0.36}{3},\\&=0.34,\\ S(L_{2})&=\frac{0.2+0.6+0.45}{3},\\&=0.42,\\ S(L_{3})&=\frac{0.55+0.36+0.47}{3},\\&=0.46. \end{aligned}$$

The rank of the alternatives based on the score functions is

$$\begin{aligned} S(L_{1})<S(L_{2})<S(L_{3}) \end{aligned}$$

As a result, the score functions based on complex fuzzy Normalized Euclidean DMs choose the Amazone $(L_{3})$ as the best choice.

**Advantages of complex fuzzy distance measures with hesitance values**

Complex fuzzy distance measures with hesitance values offer a powerful framework for tackling and modeling uncertainties in decision-making. By providing a flexible and robust approach to representing uncertain preferences, these measures enable decision-makers to make more informed and confident decisions in complex and uncertain decision environments. Hesitance values in complex fuzzy distance measures allow decision-makers to explicitly quantify their uncertainty or hesitancy towards different alternatives. By assigning hesitance values to elements of fuzzy sets, decision-makers can convey the degree to which they are uncertain about the membership of these elements, providing a more precise representation of uncertainty. Moreover, Complex fuzzy distance measures with hesitance values offer a flexible framework for representing uncertain preferences. Decision-makers can express varying degrees of uncertainty for different elements or criteria, enabling them to capture the complex and multifaceted nature of uncertainty in decision-making problems.

## Conclusion

The applications of CFDMs in decision-making algorithms are diverse and impactful, spanning various domains where decision-makers grapple with uncertainty, imprecision, and complex relationships among alternatives. In this thesis, we introduced CFHDM and CFEHDM. They are generalizations of CFNHDM and CFEDM can be added by adding the hesitance value. The ability to consider membership and hesitancy values in evaluating alternatives has proven instrumental in handling real-world complexities. Some new operations and primary results are discussed in the environment of proposed CFDMs and complex fuzzy operations. Moreover, we developed a decision-making algorithm based on CFHDM and CFEHDM. The expected limitations of complex fuzzy hesitance distance measures include computational complexity, hindering their application in real-time or resource-constrained settings, and challenges in interpretability due to intricate mathematical computations. These measures often require tuning multiple parameters, leading to sensitivity and subjectivity in parameter selection.

In the future, the proposed complex fuzzy hesitance distance measures may discuss applications in various domains where handling uncertainty, imprecision, and complexity in data relationships is crucial. This can include fields such as pattern recognition, image processing, data mining, and decision support systems. Moreover, we will extend the elaborated work for complex fuzzy intuitionistic fuzzy sets, complex fuzzy Pythagorean fuzzy sets and complex fuzzy neutrosophic sets in decision-making problems to improve the quality of the research works.

### Ethical approval

This article does not contain any studies with human participants or animals performed by any of the authors.

## Data Availability

The datasets used and/or analyzed during the current study available from the corresponding author on reason-able request.
